# Supplementary material for: Shaping active matter from crystalline solids to active turbulence
Source: Nat Commun. 2024 Apr 3;15:2874. doi: 10.1038/s41467-024-46520-4 (PMC11258367; doi:10.1038/s41467-024-46520-4)
Supplement: Supplementary file 1 — Supplementary information [file 41467_2024_46520_MOESM1_ESM.pdf]

Supplementary Information for "Shaping active matter from crystalline solids to active turbulence"

Qianhong Yang#,[1] Maoqiang Jiang#,[2,1] Francesco Picano,[3] and Lailai Zhu[1,*]
[1]Department of Mechanical Engineering, National University of Singapore, 117575, Singapore
[2]School of Naval Architecture, Ocean and Energy Power Engineering, Wuhan University of Technology, Wuhan, Hubei, 430063, PR China
[3]Department of Industrial Engineering and CISAS "G. Colombo", University of Padova, Padova, 35122, Italy

CONTENTS

I. Supplementary Notes 2
A. Fig. 1: Numerical Setup and Diverse Collective Behaviors 2
B. Fig. 2: Phase Diagram and Theoretical Scaling 2
C. Fig. 3: Defect-mediated Melting 2
D. Fig. 4: Instability and Transition of Active Fluids 3
E. Fig. 5: Active Turbulence 3
1. Energy spectrum for point microswimmers 3
2. Energy spectrum for finite-sized microswimmers 4

II. Supplementary Methods 4
A. Governing Equations 4
B. Lattice Boltzmann Method and Immersed Boundary Method 5
C. Immersed Boundary Method (IBM) 5
1. IBM for the momentum equation 5
2. IBM for the advection-diffusion equation 6
D. Validation of The LBM-IBM Solver 6
1. Hydrodynamics alone: A 2D squirmer swimming in a closed box 6
2. Coupled hydrodynamics and solute transport: Spontaneous motion of a phoretic disk 7

III. Supplementary Discussion 8
A. Theoretical scaling for the solid-liquid phase transition 8
B. Active Wigner crystal 9
C. Interaction between two phoretic disks 10
D. Formation of arc-shaped disk chains 10
E. Two-dimensional melting 12
F. Transition to and active turbulence 13
1. Effect of finite inertia on active turbulence 13
2. Resemblance with active nematics and polar active fluids 13
G. Clustering of phoretic disks 14
H. Reconciling two experimental observations on camphor surfers 14
I. Isotropic phoretic agents (IPAs) in an unbounded domain 16

Supplementary References 17

# These authors contributed equally to this work.
* lailai_zhu@nus.edu.sg

In this document, we provide supplementary information (SI) to complement the main article. Initially, we clarify the definitions of specific variables in Sec. I. Following this, the numerical implementation and validation processes are detailed in Sec. II. Sec. III A elaborates on the theoretical prediction of the phase transition.

Section III C explores typical scenarios involving the interaction of two phoretic disk swimmers and characterizes their hydrodynamic interactions. The extension from two disks to multiple disks forming chains is discussed in Sec. III D. Additional data pertaining to two-dimensional melting are presented in Sec. III E. Subsequently, Sec. III F delves into certain subtle aspects of active turbulence.

In Sec. III G, we delineate the distinctions between the microswimmer clusters observed in our study and those documented in prior research. Sec. III H is dedicated to elucidating how our findings bridge the gap between two contrasting experimental observations on camphor surfers. Lastly, the behavior of isotropic phoretic agents (IPAs) in an unbounded domain is discussed.

Variables here are dimensionless unless otherwise mentioned. Dimensional variables indicated by $\tilde{}$ as in the main article appear in Sec. III A only.

I. SUPPLEMENTARY NOTES

Here, we present the variables not elaborated on in the main article, organizing them based on their initial appearance in the corresponding figures.

A. Fig. 1: Numerical Setup and Diverse Collective Behaviors

The root-mean-square velocity $U_{\rm rms}(t)$ of N disks is:

$$U_{\rm rms}(t) = \sqrt{\frac{1}{N}\sum_{k=1}^{N}|\mathbf{U}_k(t)|^2}, \tag{1}$$

where $\mathbf{U}_k(t)$ denotes the translational velocity of the k-th disk at time t.

We define the pair correlation function $g(\mathcal{R})$ as a function of the center-to-center distance $\mathcal{R}$ between a pair of disks:

$$g(\mathcal{R}) = \frac{\sum_{k=1}^{N}\sum_{k'\neq k}\delta_{\rm dis}\left(\mathcal{R},\Delta\mathcal{R},|\mathbf{R}_{k'}-\mathbf{R}_k|\right)}{\pi\mathcal{R}\Delta\mathcal{R}N\left(N-1\right)/L^2}, \tag{2}$$

where $\mathbf{R}_k$ is the positional vector of disk k and $\Delta\mathcal{R}$ is a sufficiently small increment of $\mathcal{R}$. Here, $\delta_{\rm dis}(\mathcal{R},\Delta\mathcal{R},\mathcal{R}')$ is the discrete Dirac delta function defined below

$$\delta_{\rm dis}(\mathcal{R},\Delta\mathcal{R},\mathcal{R}') = \begin{cases}1, & \text{if } \mathcal{R}\leq\mathcal{R}'\leq\mathcal{R}+\Delta\mathcal{R},\\ 0, & \text{otherwise.}\end{cases} \tag{3}$$

The mean square displacement (MSD) as a function of the time lag τ, can be expressed as:

$$\text{MSD}(\tau) = \left\langle\overline{|\mathbf{R}^{\rm d}(t+\tau)-\mathbf{R}^{\rm d}(t)|^2}\right\rangle, \tag{4}$$

where $\mathbf{R}^{\rm d}(t) = \int_0^t\mathbf{U}(t'){\rm d}t'$ is the total displacement of a disk at time t with respect to its initial position. Here, $\langle\bullet\rangle$ indicates averaging over all disks as will be adopted throughout this work. Also, $\overline{\overline{(\bullet)}}$ means averaging over a series of moving time windows. We note that $\mathbf{R}^{\rm d}$ is distinct from $\mathbf{R}$, where the former has not been shifted to be bounded in the periodic domain, unlike the latter.

B. Fig. 2: Phase Diagram and Theoretical Scaling

In Fig. 2 of the main article, we show the time-averaged root-mean-square velocity $\overline{U_{\rm rms}}(\phi,\text{Pe})$. Here, $\overline{(\bullet)}$ means time-averaging, a convention consistently used throughout this work.

C. Fig. 3: Defect-mediated Melting

We introduce the two-dimensional (2D) static structure factor $S(\mathbf{q})$ varying with the wave vector $\mathbf{q} = q\exp(\mathrm{i}\theta)$

$$S(\mathbf{q}) = \frac{1}{N}\overline{I(\mathbf{q})I(-\mathbf{q})}, \tag{5}$$

where

$$I(\mathbf{q}) = \sum_{k=1}^{N}\exp(\mathrm{i}\mathbf{q}\cdot\mathbf{R}_k), \tag{6}$$

and i represents the imaginary unit.

The local translational $\psi_{\mathbf{q}_0}(\mathbf{R}_k)$ and orientational order $\psi_6(\mathbf{R}_k)$ are:

$$\psi_{\mathbf{q}_0}(\mathbf{R}_k) = \exp(\mathrm{i}\mathbf{q}_0\cdot\mathbf{R}_k), \tag{7a}$$

$$\psi_6(\mathbf{R}_k) = \frac{1}{\mathcal{N}_k}\sum_{j\in\mathcal{N}_k}\exp(6\mathrm{i}\varphi_{kj}), \tag{7b}$$

where $\mathbf{q}_0$ is the wave vector at the maximum of the first diffraction peak of $S(\mathbf{q})$, and $\mathcal{N}_k$ is the number of disk k's neighbours, identified through Voronoi tessellation. Besides, φ_{kj} measures the orientation of the j-th neighbour with respect to the host, *viz.*, disk k, that is, the angle between their center-to-center displacement vector and a fixed reference orientation. In this case, we choose the basis vector $\mathbf{e}_x$ as the reference without loss of generality.

The translational or orientational correlation function $g_\alpha(\mathcal{R})$ with $\alpha\equiv\mathbf{q}_0, 6$ is given by:

$$g_\alpha(\mathcal{R}) = \frac{\overline{\sum_{k=1}^{N}\sum_{k'\neq k}\psi^*_\alpha(\mathbf{R}_{k'})\psi_\alpha(\mathbf{R}_k)\delta_{\rm dis}(\mathcal{R}-|\mathbf{R}_{k'}-\mathbf{R}_k|)}}{\overline{\sum_{k=1}^{N}\sum_{k'\neq k}\delta_{\rm dis}(\mathcal{R}-|\mathbf{R}_{k'}-\mathbf{R}_k|)}}, \tag{8}$$

where the asterisk * denotes the complex conjugate operator.

D. Fig. 4: Instability and Transition of Active Fluids

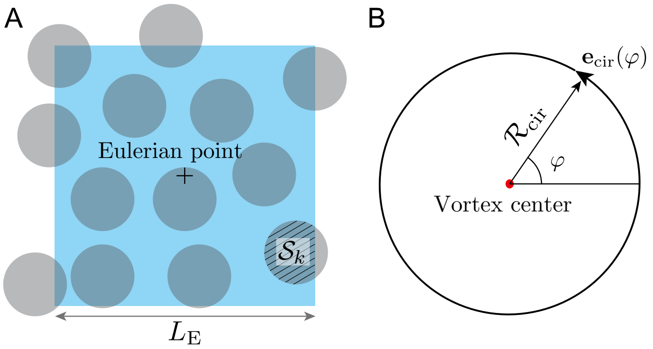

Fig. S1 : A, calculating the local variables, *e.g.*, area fraction $\phi_{\rm E}$ of disks (circles) at a given Eulerian position (+) via spatially sampling a square window. $\mathcal{S}_k$ denotes the area of disk k within the window. B, calculating the scaled circulation on a circle of radius $\mathcal{R}_{\rm cir}$. The red dot represents a candidate vortex center, φ is the polar angle, and $\mathbf{e}_{\rm cir}(\varphi)$ is the tangential unit vector.

We consider the disks as atoms or molecules of a new active matter or phase, and map the phase transition onto the classical thermodynamic version. For a fluidized active matter of such, *viz.*, active fluid manifested in Figs. 4 and 5 of the main article, we can study its continuum dynamics via an Eulerian approach in addition to the Lagrangian counterpart, *i.e.*, examining the motion of individual disks. This resembles adopting the canonical macroscopic description of fluid motion without considering the microscopic molecular motion. In the same vein, we take the macroscopic, Eulerian variable $\mathcal{V}_{\rm E}$ as the spatial average of the microscopic, Lagrangian variable $\mathcal{V}$, such as ϕ and $\mathbf{U}$ of disks.

For a given Eulerian position, we sample a square window of size $L_{\rm E}$ centred at that position (Fig. S1A) and perform area-weighted averaging of $\mathcal{V}$ to obtain

$$\mathcal{V}_{\rm E} = \frac{1}{L_{\rm E}^2}\begin{cases}\sum_k^{N_{\rm E}} \mathcal{S}_k, & \text{if } \mathcal{V} := \phi,\\ \sum_k^{N_{\rm E}} \mathcal{V}\mathcal{S}_k, & \text{otherwise.}\end{cases} \tag{9}$$

Here, $N_{\rm E}$ is the number of disks that overlap with the window, and $\mathcal{S}_k$ is the overlapping area of disk k. To ensure sufficient statistical confidence, we choose a window size $L_{\rm E} = \sqrt{10\pi/\phi}$ such that $N_{\rm E} \approx 10$ on average. Using the calculated continuum velocity $\mathbf{U}_{\rm E} = U_{\rm E}\mathbf{e}_x + V_{\rm E}\mathbf{e}_y$, we can also obtain the corresponding vorticity $\boldsymbol{\omega}_{\rm E} = \boldsymbol{\nabla}\times\mathbf{U}_{\rm E}$.

Likewise, we calculate the Okubo-Weiss (OW) parameter [1, 2],

$$\mathrm{OW} = (\frac{\partial U_{\rm E}}{\partial x} + \frac{\partial V_{\rm E}}{\partial y})^2 - 4\frac{\partial U_{\rm E}}{\partial x}\frac{\partial V_{\rm E}}{\partial y} + 4\frac{\partial U_{\rm E}}{\partial y}\frac{\partial V_{\rm E}}{\partial x} \tag{10}$$

to help identify the vortical structures in our system. To do so, we first locate all the Eulerian grid points where $\mathrm{OW} < 0$. Next, for each of these found points, we calculate the scaled circulation

$$\Gamma = \frac{1}{2\pi}\oint \frac{\mathbf{U}_{\rm E}}{|\mathbf{U}_{\rm E}|}\cdot \mathbf{e}_{\rm cir}(\varphi)\mathrm{d}\varphi \tag{11}$$

on a circle of radius $\mathcal{R}_{\rm cir}$ centered there (Fig. S1B). Here, φ is the polar angle, and $\mathbf{e}_{\rm cir}(\varphi) = -\cos\varphi\mathbf{e}_x + \sin\varphi\mathbf{e}_y$ is the tangential vector on the circle. We choose $\mathcal{R}_{\rm cir} = 3$, neglecting the vortical structures smaller than that size. This choice seems reasonable given the disk diameter of 2. Ideally, $|\Gamma| = 1$ for a grid point that coincides with the center of a perfect circular vortex. In practice, we consider a particular point as a vortex center when $|\Gamma| \geq 0.85$. Having probed the center of vortical structures, we further determine their occupied area $\mathcal{S}_{\rm vtx}$. For a probed center of vortex, we examine $\boldsymbol{\omega}_{\rm E}$ and OW at the Eulerian points within a circular domain of radius 20. Most of them would share the same sign of $\boldsymbol{\omega}_{\rm E}$, and those with $\mathrm{OW} < 0$ are counted for calculating the area $\mathcal{S}_{\rm vtx}$. Accordingly, we obtain the effective radius $l_{\rm vtx} = \sqrt{\mathcal{S}_{\rm vtx}/\pi}$ of the identified vortex.

The clusters of disks shown in Figs. 4F and 5C of the main article are identified by the following criterion: the surface-to-surface distance between every two neighbouring disks of a cluster is below 0.15. We then follow Ref. [3] to calculate their size distribution function $\mathcal{P}(N_{\rm clu})$,

$$\mathcal{P}(N_{\rm clu}) = \frac{N_{\rm clu}}{N}\mathcal{G}(N_{\rm clu}), \tag{12}$$

where $N_{\rm clu}$ is the number of disks constituting a cluster, and $\mathcal{G}(N_{\rm clu})$ is the number of clusters sized $N_{\rm clu}$. The distribution has been normalized to fulfil

$$\sum_{N_{\rm clu}=1}^{N}\mathcal{P}(N_{\rm clu}) = 1. \tag{13}$$

In Fig. 4**g** of the main article, we fit the calculated cluster size distribution following Ref. [4],

$$\mathcal{P}(N_{\rm clu}) = \mathcal{C}_1 N_{\rm clu}^{-\mathcal{C}_2}\exp\left(-N_{\rm clu}/N_{\rm clu}^{\dagger}\right), \tag{14}$$

where the prefactor $\mathcal{C}_1$, exponent $\mathcal{C}_2$, and cutoff size $N_{\rm clu}^{\dagger}$ are fitting parameters. The emergence of clustering can be characterized by $N_{\rm clu}^{\dagger}$, which is approximately 17 for $\mathrm{Pe} = 5$, and 155 when $\mathrm{Pe} = 10$.

E. Fig. 5: Active Turbulence

1. *Energy spectrum for point microswimmers*

This study examines active turbulence of finite-sized microswimmers, the calculation of the kinetic energy spectrum differs slightly from that for point microswimmers.

To start with, we describe how the spectrum $E(q)$ versus the wavenumber $q = 2\pi/\mathcal{R}$ is calculated for point microswimmers. First, we obtain the equal-time two-point velocity correlation function $g_{UU}(\boldsymbol{\mathcal{R}})$ following

$$g_{UU}(\boldsymbol{\mathcal{R}}) = \frac{\sum_{k=1}^{N} \sum_{k' \neq k} \mathbf{U}_{k'}(t) \cdot \mathbf{U}_k(t) \delta_{\text{dis}}[\boldsymbol{\mathcal{R}} - (\mathbf{R}_{k'} - \mathbf{R}_k)]}{\sum_{k=1}^{N} \sum_{k' \neq k} \delta_{\text{dis}}[\boldsymbol{\mathcal{R}} - (\mathbf{R}_{k'} - \mathbf{R}_k)]}. \quad (15)$$

Then, applying the Fourier transform of $g_{UU}(\boldsymbol{\mathcal{R}})$,

$$E(q) = \left\langle \frac{q}{2\pi} \int \exp(-\mathrm{i}\mathbf{q} \cdot \boldsymbol{\mathcal{R}}) g_{UU}(\boldsymbol{\mathcal{R}}) \mathrm{d}^2\boldsymbol{\mathcal{R}} \right\rangle_\theta, \quad (16)$$

where $\langle\rangle_\theta$ denotes averaging over the phase angle θ of the wave vector $\mathbf{q}$.

Instead of using Eq. (16), there is another alternative method. It lies in an angular average of $g_{UU}(\boldsymbol{\mathcal{R}})$ resulting in the one-dimensional velocity correlation function $g_{UU}(\mathcal{R}) = \langle g_{UU}(\boldsymbol{\mathcal{R}}) \rangle_\theta$ with $\mathcal{R} = |\boldsymbol{\mathcal{R}}|$. Accordingly, the energy spectrum can be obtained by [5]:

$$E(q) = q \int_0^\infty g_{UU}(\mathcal{R}) \mathcal{R}\ J_0(q\mathcal{R}) \mathrm{d}\mathcal{R}, \quad (17)$$

where J_0 is the zeroth-order Bessel function of the first kind. Notably, the second method assumes an isotropic active turbulence. Our analysis of the data reveals that the two expressions yield nearly the identical energy spectra with indiscernible differences.

2. *Energy spectrum for finite-sized microswimmers*

Eqs. (16) and (17) can be directly applied to active turbulence in point microswimmers, where the inter-swimmer distance $\mathcal{R}$ features a lower bound of zero. Similarly, they can also be used to calculate the energy spectrum of inertial turbulence, because the velocity correlation function $g_{UU}(\mathcal{R})$ is defined for any two positions in space, regardless of their proximity. Nevertheless, this scenario is altered for finite-sized swimmers, where the minimum inter-swimmer distance $\mathcal{R}$ is not zero but a finite value $\mathcal{R}_{\text{lb}}$, *viz.*, the lower bound. Consequently, $g_{UU}(\mathcal{R})$ is not defined when $\mathcal{R} \in [0, \mathcal{R}_{\text{lb}})$, which results in the abnormal energy spectrum $E(q)$ calculated from $g_{UU}(\mathcal{R})$ via Eq. (17). This anomaly implies a subtle incompatibility between the classical turbulence theory with the active turbulence in finite-sized active particles.

In fact, previous studies [6, 7] on active turbulence in finite-sized microswimmer have reported the "artifact" in $E(q)$ based on Eq. (17). Both groups overcome this artifact by shifting the correlation function $g_{UU}(\mathcal{R})$ along the $\mathcal{R}$ axis, yielding a shifted correlation function $\hat{g}_{UU}(\hat{\mathcal{R}})$ versus a modified inter-particle distance $\hat{\mathcal{R}}$. Here, $\hat{\mathcal{R}}$ effectively indicates the minimum surface-to-surface distance between two swimmers. Upon such a shift, the lower limit of $\hat{\mathcal{R}} = \mathcal{R} - \mathcal{R}_{\text{lb}}$ becomes zero, which permits using the canonical energy spectrum formula.

Despite circumventing the anomaly or artifact in the energy spectrum, this shifting approach introduces a side effect. The new velocity correlation function $\hat{g}_{UU}(\hat{\mathcal{R}})$ effectively measures the correlation of two finite-sized swimmers versus their minimum surface-to-surface distance $\hat{\mathcal{R}}$. This definition of distance differs from the center-to-center distance between, either two point swimmers showing active turbulence, or two spatial positions in inertial turbulence. Consequently, the energy spectrum calculated from $\hat{g}_{UU}(\hat{\mathcal{R}})$ features a new, 'shifted' wavenumber

$$\hat{q} = \frac{2\pi}{\hat{\mathcal{R}}} = \frac{2\pi}{\mathcal{R} - \mathcal{R}_{\text{lb}}}, \quad (18)$$

in contrast to the original one $q = 2\pi/\mathcal{R}$. In our case, the minimum center-to-center distance between swimmers is $\mathcal{R}_{\text{lb}} = 2$, *i.e.*, two radii of phoretic disks, leading to $\hat{q} = 2\pi/(\mathcal{R} - 2)$.

In this work, we have adopted the shifting approach and shown the energy spectrum $E(\hat{q})$ versus the shifted wavenumber $\hat{q}$ scaled by a characteristic wavenumber q_{c}. Here, $q_{\text{c}} = 2\pi/2 = \pi$ is defined based on the diameter 2 of disks.

II. SUPPLEMENTARY METHODS

A. Governing Equations

To address our hydrochemical problem, we adapt a massively parallel flow solver using a Lattice Boltzmann method (LBM) [8] and an immersed boundary method (IBM) [9, 10]. It is worth-noting that LBM solving hydrochemical dynamics was recently used to study suspensions of Janus colloids in a Hele-Shaw cell [11]. Besides LBM, the multi-particle collision dynamics method [12] has also been employed to investigate hydrochemical interactions within Janus collectives [13].

Using the LBM-IBM implementation, we do not solve exactly the Stokes equation but need to retain the inertia term for time marching. Namely, we solve the Navier-Stokes equation in the limit of low Reynolds (Re) number to approximate the Stokes equation. Thus, the actual non-dimensional governing equations solved by the LBM are

$$\boldsymbol{\nabla} \cdot \mathbf{u} = 0, \quad (19a)$$

$$\text{Re}\left(\frac{\partial \mathbf{u}}{\partial t} + \mathbf{u} \cdot \boldsymbol{\nabla}\mathbf{u}\right) = -\boldsymbol{\nabla}p + \boldsymbol{\nabla}^2\mathbf{u}, \quad (19b)$$

$$\frac{\partial c}{\partial t} + \mathbf{u} \cdot \boldsymbol{\nabla}c = \frac{1}{\text{Pe}}\boldsymbol{\nabla}^2 c. \quad (19c)$$

We choose $\text{Re} = 0.5$ throughout the work, and the effect of inertia is reasonably weak as shown in Sec. II D for a single disks and Sec. III F 1 for many.

The boundary conditions (BCs) for c and $\mathbf{u}$ at the surface of disks are:

$$\mathbf{n} \cdot \boldsymbol{\nabla} c = -1, \tag{20a}$$

$$\mathbf{u} = \mathbf{u}_{\text{slip}} + \mathbf{U} + \boldsymbol{\Omega} \times (\mathbf{r} - \mathbf{R}), \tag{20b}$$

where $\mathbf{n}$ is the outward unit normal vector, $\mathbf{u}_{\text{slip}} = (\mathbf{I} - \mathbf{nn}) \cdot \boldsymbol{\nabla} c$ is the slip velocity at the disk surface, $\mathbf{r}$ and $\mathbf{R}$ denote the coordinates of a specific point at the disk surface and the disk center, respectively. Here, $\mathbf{U} = U\mathbf{e}_x + V\mathbf{e}_y$ and $\boldsymbol{\Omega} = \Omega\mathbf{e}_z$ represent the translational and rotational velocities of the disk, respectively.

Besides the flow, we need to solve for the time-dependent position of disks. Assuming disks and the surrounding liquid have the same densities, we update $\mathbf{U}$ and $\boldsymbol{\Omega}$ of a disk using Newton's law of motion,

$$\pi \text{Re} \frac{\text{d}\mathbf{U}}{\text{d}t} = \mathbf{F}, \tag{21a}$$

$$\frac{1}{2} \pi \text{Re} \frac{\text{d}\boldsymbol{\Omega}}{\text{d}t} = \mathbf{L}, \tag{21b}$$

where $\mathbf{F}$ and $\mathbf{L}$ denote the hydrodynamic force and torque on the disk. Eq. S(21) recovers to the force-free $\mathbf{F} = \mathbf{0}$ and torque-free $\mathbf{L} = \mathbf{0}$ conditions at $\text{Re} = 0$ as typically adopted to calculate the velocities of Stokesian swimmers [14].

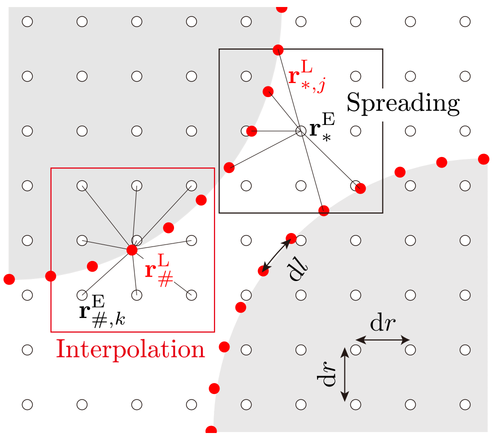

Fig. S2 : Schematic of the IBM implementation. The shaded areas indicate immersed disks. Hollow and filled circles mark Lagrangian and Eulerian grid points, respectively, with their spacings denoted by dl and dr. Here, $\mathbf{r}^{\text{E}}_{*}$ specifies a particular Eulerian point, and $\mathbf{r}^{\text{L}}_{*,j}$ refers to its j-th (out of 6 total here) neighbouring Lagrangian point. Likewise, $\mathbf{r}^{\text{L}}_{\#}$ identifies a specific Lagrangian point, and $\mathbf{r}^{\text{E}}_{\#,k}$ represents its k-th (out of 9 in total here) neighbouring Eulerian point. The square boxes illustrate the support region of a three-point Dirac delta function used for interpolation and spreading in the IBM.

B. Lattice Boltzmann Method and Immersed Boundary Method

We provide a brief introduction of the LBM-IBM solver, which is detailed in Refs. [15, 16]. We use a standard D2Q9 LBM with a single relaxation time to solve for the velocity field $\mathbf{u}$ and solute concentration c. Besides the distribution functions for the fluid density and velocity, we introduce another set of distribution functions $\{f_1, ..., f_9\}$ for c.

As shown in Fig. S2, we adopt a uniform lattice grid (hollow circles) to discretize the Eulerian fluid domain and a uniform distribution of Lagrangian points (solid circles) to represent the disk edges. The spacings of the two grids are $\text{d}r = 1/n_{\text{rad}}$ and $\text{d}l \sim O(\text{d}r)$, respectively, where n_{rad} is the number of lattices spanned by the disk radius. We choose $n_{\text{rad}} = 15$ throughout this work following a grid dependence study detailed in Sec. II D.

For LBM, the relaxation time $\sqrt{3}n_{\text{rad}}\text{Ma}/\text{Re} + 0.5$ for the fluid motion determines the time step dt. Here, $\text{Ma} = \sqrt{3}n_{\text{rad}}\text{d}t$ indicates the artificial compressibility of the LBM implementation, which should vanish in the Stokes flow. We choose $\text{Ma} = \sqrt{3}/750$ that reconciles acceptable computational costs and respecting the $\text{Ma} = 0$ limit of interest.

C. Immersed Boundary Method (IBM)

1. *IBM for the momentum equation*

We employ a direct-forcing IBM [15, 16] to impose the velocity BC Eq. S(20b) at the disk surface. The presence of the immerse boundary is accounted numerically by introducing a source term $\boldsymbol{\mathcal{F}}(\mathbf{r}^{\text{E}}, t)$ into the momentum equation Eq. S(19b). The modified equation takes the form of

$$\text{Re}\left[\frac{\partial \mathbf{u}\left(\mathbf{r}^{\text{E}}, t\right)}{\partial t} + \mathbf{u}\left(\mathbf{r}^{\text{E}}, t\right) \cdot \boldsymbol{\nabla} \mathbf{u}\left(\mathbf{r}^{\text{E}}, t\right)\right] = -\boldsymbol{\nabla} p\left(\mathbf{r}^{\text{E}}, t\right) + \boldsymbol{\nabla}^2 \mathbf{u}\left(\mathbf{r}^{\text{E}}, t\right) + \boldsymbol{\mathcal{F}}\left(\mathbf{r}^{\text{E}}, t\right). \tag{22}$$

We now explain how to calculate the source term $\boldsymbol{\mathcal{F}}$ at a specific Eulerian point $\mathbf{r}^{\text{E}}_{*}$, see Fig. S2. We first identify its neighbouring Lagrangian points $\mathbf{r}^{\text{L}}_{*,j}$ (j is the index of the neighbours) within the compact support of a three-point Dirac delta function $D(\mathbf{r}^{\text{E}}_{*} - \mathbf{r}^{\text{L}}_{*})$. Here, $D(\hat{\mathbf{r}})$ is defined by

$$D(\hat{\mathbf{r}}) = \frac{1}{\text{d}r^2} \hat{\delta}\left(\frac{|\hat{\mathbf{r}} \cdot \mathbf{e}_x|}{\text{d}r}\right) \hat{\delta}\left(\frac{|\hat{\mathbf{r}} \cdot \mathbf{e}_y|}{\text{d}r}\right), \tag{23}$$

where $\hat{\delta}(r)$ is a three-point regularized delta function [15],

$$\hat{\delta}(r) = \begin{cases} 0, & r > 1.5, \\ \frac{1}{6}(5 - 3r - \sqrt{1 - 3(1-r)^2}), & 0.5 < r \leq 1.5, \\ \frac{1}{3}(1 + \sqrt{1 - 3r^2}), & r \leq 0.5. \end{cases}$$

For the Eulerian point $\mathbf{r}_*^{\rm E}$, we calculate an intermediate velocity at all its neighboring Lagrangian points. For example, at a particular Lagrangian point $\mathbf{r}_\#^{\rm L}$, we can obtain its intermediate velocity $\mathbf{u}^{\rm int}(\mathbf{r}_{\#,j}^{\rm L},t)$ via interpolation,

$$\mathbf{u}^{\rm int}(\mathbf{r}_\#^{\rm L},t)=\sum_k \mathbf{u}^{\rm int}(\mathbf{r}_{\#,k}^{\rm E},t)D\left(\mathbf{r}_{\#,k}^{\rm E}-\mathbf{r}_\#^{\rm L}\right){\rm d}r^2, \quad (24)$$

where $\mathbf{r}_{\#,k}^{\rm E}$ denotes the k-th (out of all) neighbouring Eulerian point of $\mathbf{r}_\#^{\rm L}$.

Having known $\mathbf{u}^{\rm int}(\mathbf{r}_{*,j}^{\rm L},t)$, we compute the force per unit volume $\mathbf{f}(\mathbf{r}_{*,j}^{\rm L},t)$ acting on the Lagrangian point. It is determined, at each time step, by considering the difference between the velocity at $\mathbf{r}_{*,j}^{\rm L}$ prescribed by Eq. S(20b) and the intermediate one,

$$\mathbf{f}(\mathbf{r}_{*,j}^{\rm L},t)=\frac{2{\rm Re}}{{\rm d}t}[\mathbf{u}(\mathbf{r}_{*,j}^{\rm L},t)-\mathbf{u}^{\rm int}(\mathbf{r}_{*,j}^{\rm L},t)]. \quad (25)$$

The total hydrodynamic force $\mathbf{F}$ and torque $\mathbf{L}$ acting on the disk are then determined based on the force density of all its Lagrangian points,

$$\mathbf{F}=\sum_j \mathbf{f}(\mathbf{r}_{*,j}^{\rm L},t){\rm d}l{\rm d}r, \quad (26a)$$

$$\mathbf{L}=\sum_j (\mathbf{r}_{*,j}^{\rm L}-\mathbf{R})\times\mathbf{f}(\mathbf{r}_{*,j}^{\rm L},t){\rm d}l{\rm d}r. \quad (26b)$$

Subsequently, we employ the forward Eulerian scheme to solve Eq. S(21) and update the translational $\mathbf{U}$ and rotational $\mathbf{\Omega}$ velocities of the disk by using $\mathbf{F}$ and $\mathbf{L}$, respectively.

Finally, at the Eulerian point $\mathbf{r}_*^{\rm E}$, we determine $\boldsymbol{\mathcal{F}}(\mathbf{r}_*^{\rm E},t)$ by spreading $\mathbf{f}(\mathbf{r}_{*,j}^{\rm L},t)$ from its neighboring Lagrangian points

$$\boldsymbol{\mathcal{F}}(\mathbf{r}_*^{\rm E},t)=-\sum_j \mathbf{f}(\mathbf{r}_{*,j}^{\rm L},t)D\left(\mathbf{r}_{*,j}^{\rm L}-\mathbf{r}_*^{\rm E}\right){\rm d}l{\rm d}r. \quad (27)$$

2. *IBM for the advection-diffusion equation*

Besides the velocity, a direct-forcing IBM is also applied for the flux BC Eq. S(20a). It is achieved through another direct-forcing IBM specifically designed for thermal problems [17]. The more general form of Eq. S(20a) reads

$$\mathbf{n}\cdot\boldsymbol{\nabla}c(\mathbf{r}^{\rm L},t)=-m(\mathbf{r}^{\rm L},t){\rm Pe}/{\rm d}r, \quad (28)$$

which includes Eq. S(20a) as a special case of $m={\rm d}r/{\rm Pe}$. The essential idea for imposing a flux on immersed boundaries is to determine, at every time step, a source term $M(\mathbf{r}^{\rm E},t)$ introduced in Eq. S(19c). The modified equation is

$$\begin{aligned}\frac{\partial c\left(\mathbf{r}^{\rm E},t\right)}{\partial t}&+\mathbf{u}\left(\mathbf{r}^{\rm E},t\right)\cdot\boldsymbol{\nabla}c\left(\mathbf{r}^{\rm E},t\right)\\ &=\frac{\boldsymbol{\nabla}^2c\left(\mathbf{r}^{\rm E},t\right)}{\rm Pe}+M\left(\mathbf{r}^{\rm E},t\right). \quad (29)\end{aligned}$$

Calculating $c(\mathbf{r}^{\rm E},t)$ at Eulerian points starts with an intermediate prediction $c^{\rm int}(\mathbf{r}^{\rm E},t)=\sum_i f_i(\mathbf{r}^{\rm E},t)$ without satisfying the BC. Then, the predicted value is compensated by a correction $c^{\rm c}(\mathbf{r}^{\rm E},t)=M(\mathbf{r}^{\rm E},t){\rm d}t/2$ proportional to the source term [18], namely $c(\mathbf{r}^{\rm E},t)=c^{\rm int}(\mathbf{r}^{\rm E},t)+c^{\rm c}(\mathbf{r}^{\rm E},t)$.

We now illustrate the procedure for calculating the source term M at a specific Eulerian point $\mathbf{r}_*^{\rm E}$. After identifying its neighboring Lagrangian points $\mathbf{r}_{*,j}^{\rm L}$, we compute the corresponding intermediate concentration flux

$$m^{\rm int}(\mathbf{r}_{*,j}^{\rm L},t)=-\mathbf{n}\cdot\boldsymbol{\nabla}c^{\rm int}(\mathbf{r}_{*,j}^{\rm L},t){\rm d}r/{\rm Pe}, \quad (30)$$

where $\boldsymbol{\nabla}c^{\rm int}(\mathbf{r}_{*,j}^{\rm L},t)$ is the intermediate concentration gradient at $\mathbf{r}_{*,j}^{\rm L}$. Likewise, at a particular Lagrangian point $\mathbf{r}_\#^{\rm L}$, we can obtain this gradient $\boldsymbol{\nabla}c^{\rm int}$ via interpolation,

$$\boldsymbol{\nabla}c^{\rm int}(\mathbf{r}_\#^{\rm L},t)=\sum_k\boldsymbol{\nabla}c^{\rm int}(\mathbf{r}_{\#,k}^{\rm E},t)D\left(\mathbf{r}_{\#,k}^{\rm E}-\mathbf{r}_\#^{\rm L}\right){\rm d}r^2. \quad (31)$$

Upon acquiring $m^{\rm int}(\mathbf{r}_{*,j}^{\rm L},t)$, we compute the difference

$${\rm d}m(\mathbf{r}_{*,j}^{\rm L},t)=m(\mathbf{r}_{*,j}^{\rm L},t)-m^{\rm int}(\mathbf{r}_{*,j}^{\rm L},t)$$

between the expected value $m(\mathbf{r}_{*,j}^{\rm L},t)$ and the intermediate one. Finally, we determine $M(\mathbf{r}_*^{\rm E},t)$ by spreading the flux differences from its neighboring Lagrangian points

$$M(\mathbf{r}_*^{\rm E},t)=\sum_j 2{\rm d}m(\mathbf{r}_{*,j}^{\rm L},t)D\left(\mathbf{r}_{*,j}^{\rm L}-\mathbf{r}_*^{\rm E}\right){\rm d}l. \quad (32)$$

D. Validation of The LBM-IBM Solver

1. *Hydrodynamics alone: A 2D squirmer swimming in a closed box*

Focusing on a single active particle, we validate our LBM-IBM solver against theory and/or numerical data obtained via finite element method (FEM). The FEM implemented in COMSOL Multiphysics (I-Math, Singapore) uses a body-fitted mesh other than the diffused interface of IBM, which thus guarantees the accuracy of benchmark solutions. The validation is performed for two benchmark settings: 1) a model microswimmer with prescribed surface actuation swimming in a box, and 2) the spontaneous propulsion of an isotropic phoretic disk; the former involves hydrodynamics alone and the latter features coupled hydrodynamics and solute transport.

We consider the locomotion of a circular model microswimmer moving in a 2D closed box. Our model swimmer, the widely studied squirmer, was initially proposed to mimic ciliated microorganisms [19, 20]. The 2D squirmer self-propels using a surface actuation symmetric about its orientation $\mathbf{e}$. This actuation is represented by a tangential slip velocity $\mathbf{u}_{\rm slip}(\vartheta)$ distributed along the

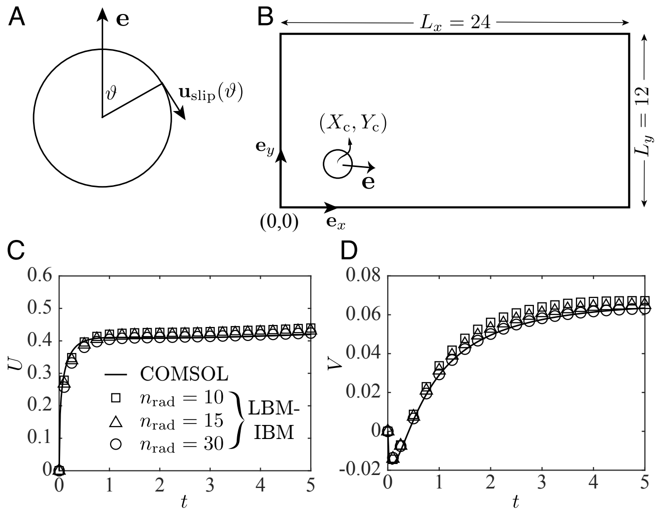

Fig. S3 : A, A 2D squirmer self-propels by its surface actuation represented by a slip velocity $\mathbf{u}_{\rm slip}(\vartheta)$ symmetric about its orientation $\mathbf{e}$. Here, ϑ is the polar angle at the surface with respect to $\mathbf{e}$. B, Locomotion of a squirmer in a closed box with length $L_x = 24$ and height $L_y = 12$. The squirmer centered at (X_c, Y_c) swims at a velocity $\mathbf{U} = U\mathbf{e}_x + V\mathbf{e}_y$. C and D, Velocity components U and V evolve over time t; the curve and symbols correspond to the COMSOL and LBM-IBM data, respectively.

polar angle ϑ with respect to $\mathbf{e}$, as shown in Fig. S3A. We adopt a common velocity distribution

$$\mathbf{u}_{\rm slip} = (\sin\vartheta + \beta \sin\vartheta \cos\vartheta)\,\mathbf{e}_\vartheta, \tag{33}$$

including the first two squirming modes [19]. Here, β indicates the ratio of the second mode to the first mode. We choose the first mode as the characteristic velocity rather than $\mathcal{AM}/\mathcal{D}$ of the hydrochemical problem, leaving the resulting dimensionless equations for fluid motion unchanged.

Using COMSOL and LBM-IBM, we solve Eqs. (19a), (19b) and (21) for the setting depicted in Fig. S3B. The box's length and height are $L_x = 24$ and $L_y = 12$, respectively, with the origin $(x, y) = (0, 0)$ at its left-bottom corner. No-slip BCs are imposed on all four sides. At $t = 0$, the squirmer, centered at $(X_c, Y_c) = (3, 4)$, is oriented slightly towards the bottom wall; the angle between its orientation $\mathbf{e}$ and the $\mathbf{e}_x$ axis is 5°. We choose $\beta = 2$ and Re $= 0.5$ for this study.

We first obtain the benchmark solution from COMSOL. Approximate 10^5 Taylor-Hood elements are used for discretization, allowing us to eliminate the unnecessary streamwise diffusion invoked by default. The mesh is refined near the disk, with the smallest and largest elements sized 0.025 and 0.25, respectively. In the frame of COMSOL, we adopt the Global Equations node to solve Eq. S(21) and the Moving Mesh Interface to handle the time-evolving domain.

We conduct LBM-IBM simulations with three grid resolutions featuring $n_{\rm rad} = 10, 20,$ and 30. Increasing this resolution yields better agreement between the LBM-IBM and COMSOL data (see Fig. S3C-D). Their excellent matching when $n_{\rm rad} = 30$ suggests that both solvers have been cross-validated, considering their completely different numerical algorithms. Notably, our COMSOL implementation for diverse chemically phoretic swimmers, including disks and particles, has undergone thorough validation, as detailed in Ref. [21].

2. *Coupled hydrodynamics and solute transport: Spontaneous motion of a phoretic disk*

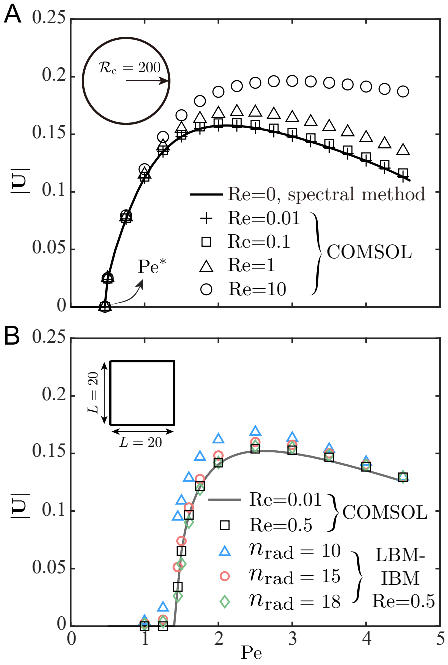

Fig. S4 : A two-step approach to validate the LBM-IBM implementation. A, firstly, developing a COMSOL solver firmly validated against a spectral code [22] for a spontaneously swimming phoretic disk in a circular domain of radius $\mathcal{R}_c = 200$. B, secondly, validating the LBM-IBM approach against the COMSOL solver developed in the first step for a spontaneously swimming phoretic disk in a square domain sized $L = 20$. More details can be found in the text.

Having validated our LBM-IBM solver for the pure hydrodynamic problem, we turn to the hydrochemical scenario of an isotropic phoretic disk, mathematically described in Sec. II A. For a single disk centered in a circular fluid domain, the critical Péclet number Pe* corresponding to the onset of instability has been derived theoretically by Ref. [22] in the creeping flow regime, *viz.*, Re $= 0$ (inertialess limit); the authors also used a

Chebyshev spectral method to calculate the disk's swimming velocity for various unstable Pe values. A Dirichlet BC $c = 0$ is imposed at the outer boundary of the domain.

Considering the inconvenience of discretizing a curved domain with LBM, we adopt a two-step approach to validate our LBM-IBM solver: first, we develop a COMSOL implementation convincingly validated against Ref. [22] for a disk in a circular domain of radius $\mathcal{R}_\mathrm{c}$ (see Fig. S4A); second, we use this FEM solver to benchmark the LBM-IBM counterpart for a disk in a square domain sized L. Here, we exploit the flexibility of FEM in discretizing complex domains and its intact validity subject to domain variation.

In the first step using COMSOL alone, we solve the flow in the frame of the disk. Hence, Dirichlet BCs $\mathbf{u} = -\mathbf{U}$ and $c = 0$ are specified at the outer edge of the domain. The undeformed ring-shaped fluid domain is discretized by approximately 32000 triangular elements. Besides the Taylor-Hood discretization for the flow equation as in Sec. II D 1, the elements are of second order to represent the concentration c. The mesh is refined near the disk and enlarged in the far field, with sizes of the smallest and largest elements being 0.05 and 9.24, respectively. We choose a specific domain size $\mathcal{R}_\mathrm{c} = 200$ to compare our results with those of Ref. [22]. In addition to the inertialess limit Re $= 0$ [22], we vary Re $\in [0.01, 10]$ to probe the effect of inertia as a by-product of the validation. Moreover, Pe is limited to the range when the disk is stationary or swimming steadily. As shown in Fig. S4A, the disk's swimming speed $|\mathbf{U}|$ at different Re from COMSOL simulations agrees well with the prediction of Ref. [22] near the onset of instability. Inertia plays a negligible role in this regime. Additionally, COMSOL results lie on top of the benchmark solution (Re $= 0$) for all Pe values when Re $= 0.01$. The speed is observed to increase with Re, becoming more pronounced at larger Pe. When Pe $= 4.5$, the speed at Re $= 10$ is larger than its inertialess limit by about 65%.

Now we use the validated COMSOL solver to benchmark our LBM-IBM implementation, targeting a phoretic disk in a square domain of size $L = 20$ (Fig. S4B). The COMSOL setup differs from that in the first step (Fig. S4A) only in the shape and size of the fluid domain, thus the equations are also solved in the body frame with unchanged BCs. On the other hand, the LBM-IBM simulations are conducted in the lab frame, with the corresponding BCs $\mathbf{u} = \mathbf{0}$ and $c = 0$ imposed at the four sides of the domain. In COMSOL, the disk remains in the domain center and is equidistant from the four boundaries. For a reliable comparison, the same effect of boundaries is realized in LBM based on the moving-grid technique [23]. It is important to note that this technique is used only for validation purposes here but not for the cases of suspensions in a periodic domain. Using COMSOL, we calculate the Pe-dependent swimming speed $|\mathbf{U}|$ for Re $= 0.01$ and Re $= 0.5$. The small difference between the two datasets suggests a reasonably weak inertia effect at Re $= 0.5$, which can be chosen to approximate the Stokes flow of interest. Subsequently, $|\mathbf{U}|\,(\mathrm{Pe})$ at Re $= 0.5$ is computed with LBM-IBM using three grid resolutions $n_\mathrm{rad} = 10$, 15, and 18. As expected, increasing the resolution n_rad produces better agreement between the COMSOL and LBM-IBM data. Based on this agreement, we believe that a combination of Re $= 0.5$ and $n_\mathrm{rad} = 15$ achieves an appropriate balance between computational cost and precision for simulating Stokesian suspensions of active microswimmers. A reduced Re and increased resolution will be adopted in our future studies.

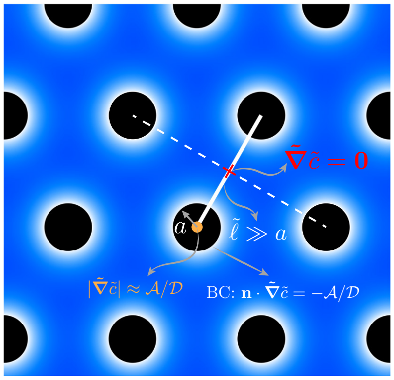

Fig. S5 : Schematic for better explaining the scaling theory. Black circles denote the disks forming a hexagonal lattice. The text provides more details.

III. SUPPLEMENTARY DISCUSSION

A. Theoretical scaling for the solid-liquid phase transition

In the main article (shown in Fig. 2), we develop a scaling to predict the transition from the solid to liquid phase. In the solid phase, disks self-organize into a stationary hexagonal lattice. This base state becomes unstable when the phoretic activity Pe surpasses a certain threshold. Accordingly, the disks start to swim, leading to the liquidation of the hexagonal state.

The hexagonal base state is portrayed in Fig. S5 to help rationalize the scaling argument. The argument relies on three aspects. First, realizing the BC $\mathbf{n} \cdot \tilde{\nabla}\tilde{c} = -\mathcal{A}/\mathcal{D}$ at the disks surface and assuming a much larger inter-disk space $\tilde{\ell}$ than a in the limit of low ϕ, we can approximate $\tilde{\nabla}\tilde{c}$ at the disk center by $\mathcal{A}/\mathcal{D}$. Second, $\tilde{\nabla}\tilde{c} = \mathbf{0}$ in the middle (marked by a cross) of two neighbouring disks; indeed, the two mirror symmetries at the middle position imply that the gradients of $\tilde{c}$ in the two orthogonal directions are both strictly zero, leading to zero $\tilde{\nabla}\tilde{c}$. Third, we have implicitly assumed an infinite-fold rotational symmetry (circular symmetry) that is stronger than the actual six-fold rotational symmetry, leading to zero $\tilde{\nabla}\tilde{c}$ everywhere on the ring of radius $\tilde{\ell}/2$ centered at each disk.

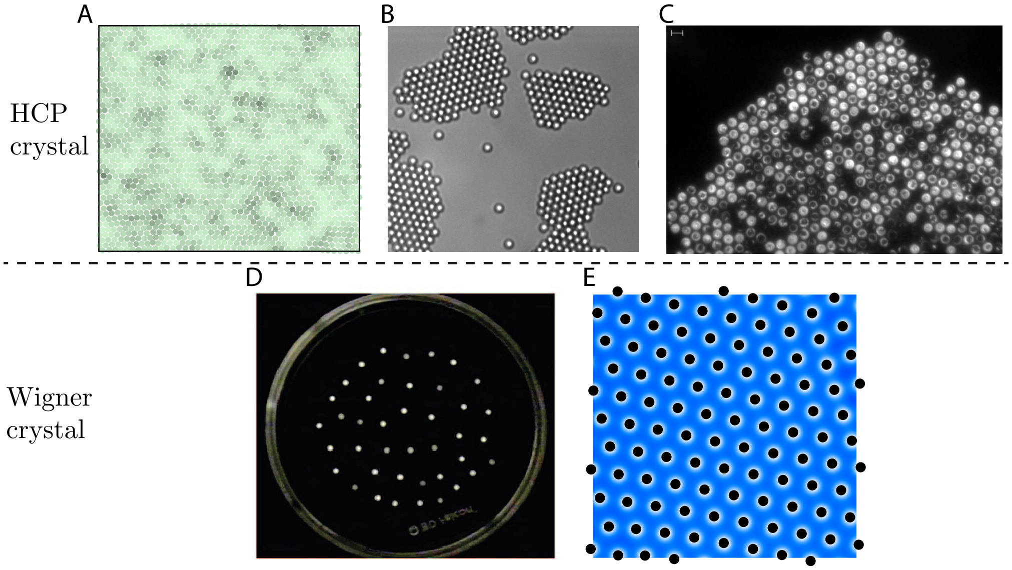

Fig. S6 : Hexagonal closed-packed crystal (upper row) and hexagonal Wigner crystal (lower row) formed in active matter systems. A, Brownian dynamics computer simulations of a Yukawa model of self-propelled particles, adapted with permission from [24]. Copyright (2012) by the American Physical Society. B, a monolayer of photoactivated Janus colloids, adapted from [25] with permission from AAAS. C, bacterial crystal formed by *Thiovulum majus*, adapted with permission from [26]. Copyright (2015) by the American Physical Society. D, Wigner crystal-like lattice formed by camphor surfers, reproduced with permission from [27]. Copyright 2008 American Chemical Society. E, Wigner crystal of isotropic phoretic agents we observe (Fig. 1**d** of the main article), with an area fraction of $\phi = 0.12$.

B. Active Wigner crystal

We provide further discussion on the hexagonal crystal formed by the phoretic disks, as exemplified by Fig. 1**d** of the main article. Our identified hexagonal structure differs from the previously observed ones formed by other active swimmers [24–26, 28–31]. Besides the different mechanisms for crystallization, an evident difference is the packing fraction (PF) of crystals.

In the above-mentioned works, the observed crystal presents a monolayer hexagonal close-packed (HCP) arrangement (see the upper row of Fig. S6); in this setting, the swimmers representing atoms pack as densely as possible, hence approaching the maximum possible PF of $\pi/\left(2\sqrt{3}\right) \approx 0.907$ [32, 33]—not to be confused with the well-known PF of $\pi/\left(3\sqrt{2}\right) \approx 0.74$ for the more general three-dimensional HCP arrangement. A graphene layer typically features PF ≈ 0.907, whereas metals like magnesium and titanium crystallize in the HCP structure with PF ≈ 0.74.

In contrast, the PF mirroring the area fraction ϕ we have defined—of our examined crystal structure can plummet to as low as 0.005. This scenario corresponds to the data point at the lower left corner in Fig. 2 of our manuscript. Such a crystalline configuration embodies what is known as the Wigner crystal, exemplified by the lower row of Fig. S6). Theoretically predicted by Eugene Wigner in 1934 [34, 35], this solid phase of electrons was only recently visualized in experiments [36]. A Wigner crystal forms at low electron densities, where the distance between electrons significantly exceeds the effective "electron size", denoted by the wave function's extent. The separation of these two length scales enables long-range Coulomb repulsion between electrons to overpower their kinetic energy, thus giving rise to an ordered lattice structure.

The formation of a Wigner crystal from our phoretic disks can be traced back to the established equivalence between phoretic and Coulomb interactions [37–39]: both exhibit repulsive characteristics and their potential decays slowly as $1/\hat{r}$ with $\hat{r}$ being the distance. Drawing parallels with the conditions for the formation of the original Wigner crystal—where the electrons' kinetic energy is significantly weaker than their repulsion—in our case of phoretic agents, they crystallize when their activity, indicated by the Pe number, falls below a certain threshold.

In addition to the collective behaviour of phoretic disks, we have also examined the pairwise interaction between two disks. As shown in Fig. S7A (see Supplementary Video 9), our simulations reproduce the typical crossing and reflecting trajectories of two phoretic swimmers previously modelled [40, 41] and experimentally observed on active droplets [41]. Here, we simulate two or three disks in a periodic domain of $L = 100$ and set Pe $= 2.5$, at which an isolated disk swims steadily. Beyond these two scenarios, we identify a stable bound

state of two disks swimming in parallel, as illustrated in Fig. S7B and Supplementary Video 10.

C. Interaction between two phoretic disks

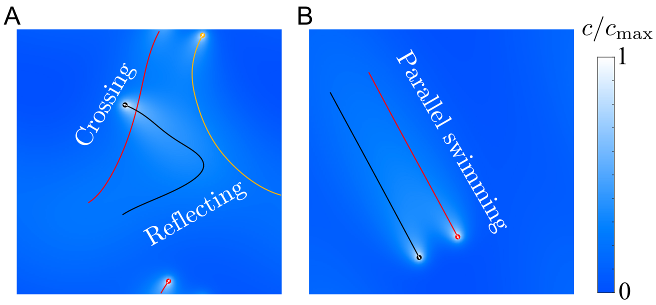

Fig. S7 : Characteristic scenarios of two interacting phoretic disks at Pe = 2.5 in a periodic domain of $L = 100$. A, crossing or reflecting trajectories of two disks. B, two disks form a stable bound pair swimming in parallel. The colormap shows the scaled concentration $c/c_{\max}$.

The crossing and reflecting scenarios can be captured by models without considering hydrodynamics. However, the stable parallel state has only been discovered in experiments naturally involving hydrodynamics and hydrodynamic interactions (HIs), as reported very recently [42]. In contrast, it has not been captured by modelling before. Considering this difference and the state of two bound swimmers reproduced by our hydrodynamic simulations, we speculate that this emerging state is related to their HI, as analyzed below.

We scrutinize the velocity evolution of the two disks numbered I (red trajectory) and II (black trajectory) in Fig. S7B. To focus on their relative motion, we define a local frame $\mathbf{e}_{\mathcal{XY}}$ attached to the center of disk II, as shown in Fig. S8A. Here, $\mathbf{e}_{\mathcal{Y}}$ is aligned with its swimming direction, and rotating $\mathbf{e}_{\mathcal{Y}}$ clockwise by 90° results in $\mathbf{e}_{\mathcal{X}}$. We characterize the emergence of the parallel bound swimming state by the time evolution of the disks' rotational velocity velocities Ω, as well as their local velocity components $\mathbf{U}^{(\mathrm{I,II})} \cdot \mathbf{e}_{\mathcal{X}}$ and $\mathbf{U}^{(\mathrm{I,II})} \cdot \mathbf{e}_{\mathcal{Y}}$. After entering their swimming states, the two swimmers initially depart ($\mathbf{U}^{(\mathrm{I})} \cdot \mathbf{e}_{\mathcal{X}} < 0$) and then attract each other ($\mathbf{U}^{(\mathrm{I})} \cdot \mathbf{e}_{\mathcal{X}} > 0$), as indicated by Fig. S8C. Following periodic switches between the two states with a decaying strength, the swimmers eventually form a parallel bound pair with the swimming velocity $\mathbf{U} \cdot \mathbf{e}_{\mathcal{Y}}$ (see Fig. S8D). Meanwhile, the two disks rotate in opposite directions at a magnitude negligible compared to their translational speed (see Fig. S8B).

The departing behaviour ($\mathbf{U}^{(\mathrm{I})} \cdot \mathbf{e}_{\mathcal{X}} < 0$ in Fig. S8C) of the two disk swimmers is known to result from their chemo-repulsive interaction [41, 43]. On the other hand, their attraction ($\mathbf{U}^{(\mathrm{I})} \cdot \mathbf{e}_{\mathcal{X}} > 0$) is attributed to the inter-swimmer HI, as evidenced experimentally for chemically active droplets [42]. These droplet swimmers are recognized to behave like a pusher-type squirmer [41, 44] (see the left panel of Fig. **1c** of the main article), exhibiting a dipolar flow pattern that pulls fluid in from the sides [45]. Consequently, two swimmers will experience hydrodynamic attraction towards each other from the sides.

Here, we devise a two-step numerical approach that decouples hydrodynamics from phoretic dynamics, allowing us to focus on the role of HI resulting from the dipolar flow pattern. The simulations are conducted using the COMSOL implementation described in Sec. II D. First, we extract the slip velocity at the surface of a steadily swimming phoretic disk, where we solve the hydrochemical problem; the slip velocity is left-right symmetric about its swimming orientation. Second, we study two identical close-by disks propelled by the slip velocity extracted from the first step when addressing the hydrodynamic problem alone. Realizing the negligible disk rotation within the bound state (see Fig. S8B), we consider an instantaneous configuration of two parallel-oriented squirmer-like disks, whose connecting line is normal to their common orientation (see Fig. S9A). Exploiting the mirror symmetry of this setting, we simulate one swimming disk using a symmetry BC.

D. Formation of arc-shaped disk chains

By examining in Fig. S9 the disk's velocities U, V, and Ω versus its activity Pe $\in [1, 4]$ and the inter-disk distance $\mathcal{R}_{\mathrm{gap}} \in (2, 20]$, we highlight the HI between two disks. At short distances, the HI enhances the swimming velocity V. Besides, it drives the disk to rotate at a negative Ω, moving away from the its compeer—the mirror disk not shown in Fig. S9A. $|\Omega|$ decreases with $\mathcal{R}_{\mathrm{gap}}$ and becomes insignificant at an approximate threshold $\mathcal{R}_{\mathrm{gap}} \approx 5$, consistent with Fig. S8B. Above this threshold, *i.e.*, $\mathcal{R}_{\mathrm{gap}} \gtrapprox 5$, our results align with the assumed parallel configuration. Hence, the following analysis on the HI will be limited to the range $\mathcal{R}_{\mathrm{gap}} \gtrapprox 5$.

The negative U shown in Fig. S9D evidences the HI-induced attraction between disks, and the inset illustrating its magnitude $|U| \propto \mathcal{R}_{\mathrm{gap}}^{-1}$ indicates the long-range nature of the hydrodynamic attraction. We further characterize the attractive strength by $|U/V|$ (Fig. S9E), which is observed to scale with the phoretic activity Pe as $|U/V| \propto \mathrm{Pe}^{5/4}$. Considering $|U| \propto \mathcal{R}_{\mathrm{gap}}^{-1}$ and the weak dependence of V on $\mathcal{R}_{\mathrm{gap}}$ (Fig. S9C), $|U/V| \propto \mathcal{R}_{\mathrm{gap}}^{-1}$ holds. Taken together, we infer that

$$|U/V| \propto \mathcal{R}_{\mathrm{gap}}^{-1} \mathrm{Pe}^{\frac{5}{4}} \tag{34}$$

as evidenced in Fig. S9F, which hints that the hydrodynamic attraction becomes stronger with increasing Pe and weakens with a larger inter-disk distance.

We observe formation of disk chains in gas-like phases when the area fraction $\phi < 0.3$, akin to the exper-

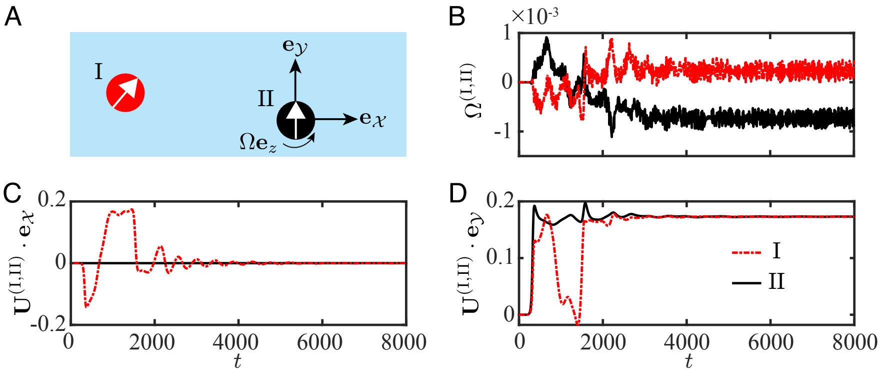

Fig. S8 : A, schematic of two interacting disks numbered I (red) and II (black). B, time evolution of their rotational velocities $\Omega^{(\mathrm{I})}$ and $\Omega^{(\mathrm{II})}$. C and D, similar to B, but for the velocity components $\mathbf{U}^{(\mathrm{I,II})}\cdot\mathbf{e}_{\mathcal{X}}$ and $\mathbf{U}^{(\mathrm{I,II})}\cdot\mathbf{e}_{\mathcal{Y}}$ in the local frame $\mathbf{e}_{\mathcal{XY}}$, respectively.

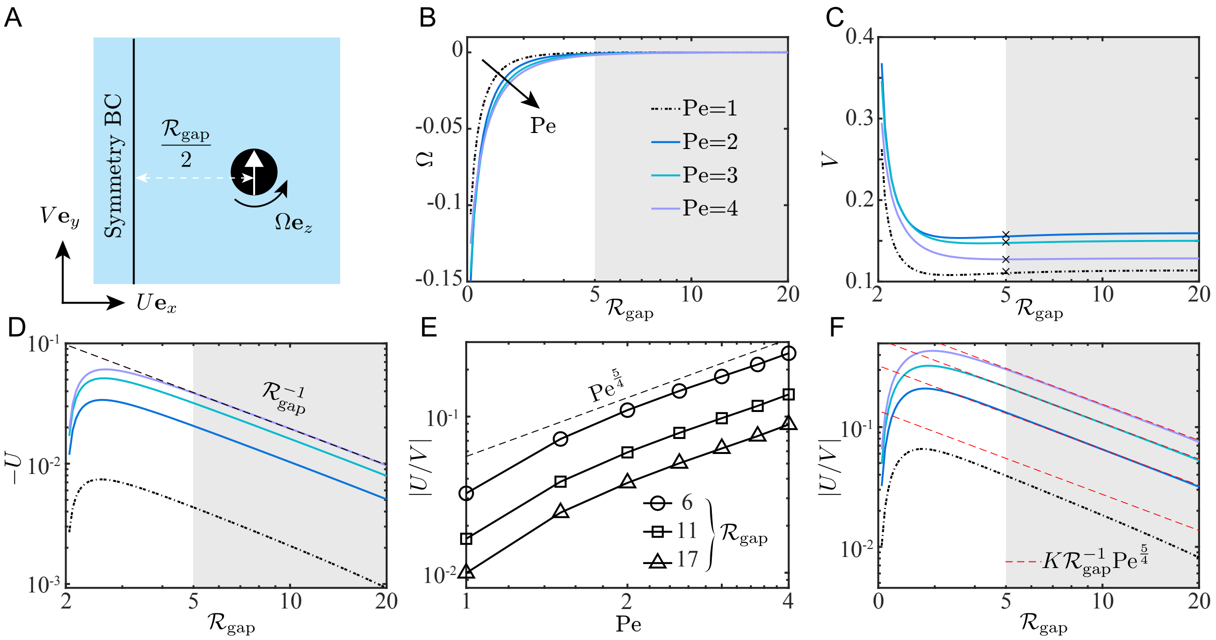

Fig. S9 : A, numerical setup for examining the HI among two swimming phoretic disks separated by $\mathcal{R}_{\mathrm{gap}}$. A symmetry BC is used here. The text provides detailed information on the setup. B-D, the disk's rotational velocity Ω, and translational velocity components V and $-U$, respectively, versus the inter-disk distance $\mathcal{R}_{\mathrm{gap}}$ at varying $\mathrm{Pe}\in[1,4]$. The shaded area indicates the regime in which the results are considered consistent with the adopted assumption of negligible disk rotation. The crosses in C represent the swimming speed of the corresponding isolated disks. A log-log scale is adopted in D. E, $|U/V|$ quantifying the HI-induced attraction between disks versus Pe at varying distances. Numerical data (symbols) are compared to the fitted curves. F, fitting $|U/V|\,(\mathrm{Pe},\mathcal{R}_{\mathrm{gap}})$ by $K\mathcal{R}_{\mathrm{gap}}^{-1}\mathrm{Pe}^{5/4}$ with $K\approx1.5$.

imentally observed counterparts of chemically active droplets [46, 47] (Fig. S10). Furthermore, this scenario can be divided into two categories depending on ϕ: at an intermediate fraction, $0.05\lessapprox\phi\lessapprox0.25$, disk chains form, colloid, disappear, and re-form; this phenomenon is termed dynamic chaining in the main article (Fig. 1**f** and Supplementary Video 2); in the dilute regime, $\phi=0.005$, a single chain swims stably as a whole. The stable chain formation is symbolized by squares in the phase diagram—Fig. 2 of the main article.

A single disk chain can be regarded as an extension of two HI-bound swimmers discussed earlier to multiple ones. Accordingly, the implication of Eq. S(34) may apply analogously: increasing the number N of disks (decreasing the inter-disk gap $\mathcal{R}_{\mathrm{gap}}$ effectively) or the activity Pe enhances the hydrodynamic attraction, thereby facilitating chain formation.

This analogy has been qualitatively confirmed as

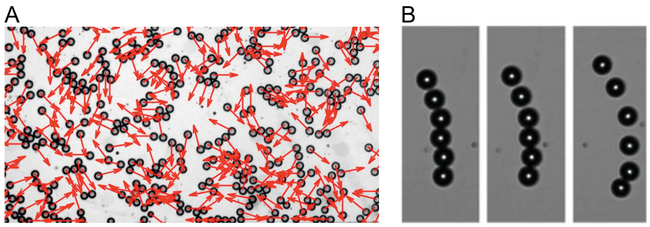

Fig. S10 : Chain formation of chemically active droplets observed in experiments. A is reproduced from Ref. [46] by permission of IOP Publishing under the CC BY-NC-SA licence. © Deutsche Physikalische Gesellschaft. B is reproduced from Ref. [47] with permission from the National Academy of Sciences (NAS).

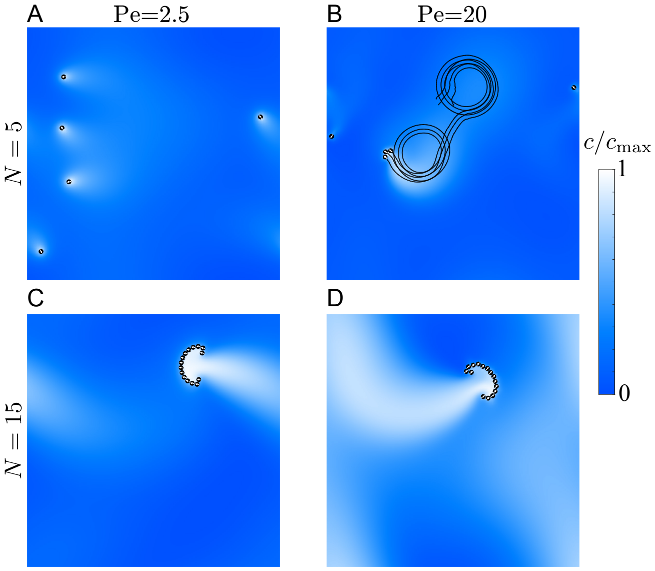

Fig. S11 : Collective behaviors of $N = 5$ (top row) and $N = 15$ (bottom row) disks in a periodic domain of $L = 100$, when Pe $= 2.5$ (left column) and Pe $= 20$ (right column). A, freely swimming disks without forming a specific pattern. B, a three-disk chain develops, executing circular or straight trajectories intermittently, see Supplementary Video 11. C and D, an arc-shaped chain forms. The colormap shows the scaled concentration $c/c_{\max}$.

demonstrated below. We investigate $N \in [3, 15]$ disks at Pe $= 2.5$ and Pe $= 20$ in a periodic domain sized $L = 100$. The chain formation begins when N reaches 5. When $N = 5$, no chain forms at Pe $= 2.5$ (Fig. S11A). The formation occurs at the higher activity Pe $= 20$. The resulting three-disk chain alternates between circular (Fig. S11B) and straight motion intermittently, leaving a solute trail in its wake (Supplementary Video 11). When $N = 15$, an arc-shaped chain emerges at both Pe values (Fig. S11C-D).

It is noteworthy that similar chain formations have also been observed in other colloidal systems due to magnetic or electric dipolar interactions among colloids [48, 49]. For instance, in Ref. [48], head-to-tail chains of dipole-like colloids were reproduced using Discontinuous Molecular Dynamics simulations. A comparison between the two chain formations is given in Tab. S1.

E. Two-dimensional melting

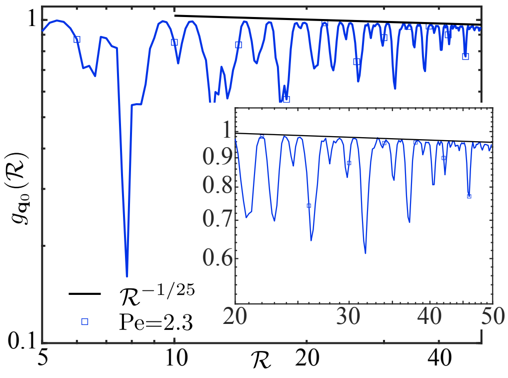

Fig. S12 : Translational order correlation function $g_{\mathbf{q}_0}(\mathcal{R})$ for Pe $= 2.3$. The inset enlarges the scaling behavior in the large $\mathcal{R}$ regime.

In the main article (Fig. 3), we investigate the melting experienced by a disk suspension of area fraction $\phi = 0.12$ as the effective temperature Pe increases. At Pe $= 2.3$, the translational order correlation function $g_{\mathbf{q}_0}(\mathcal{R}) \propto \mathcal{R}^{-\eta}$ with $\eta \approx 1/25$. Here, we provide a clearer view of this scaling behavior in Fig. S12.

Importantly, we have examined whether and how the melting dynamics depend on domain size L. We perform expanded simulations with a larger size $L = 200$ against the $L = 100$ used previously. We show the dependencies of the translational $g_{\mathbf{q}_0}(\mathcal{R})$ and orientational $g_6(\mathcal{R})$ correlation functions on L. Fig. S13 depicts the dependency of the translational order correlation function $g_{\mathbf{q}_0}(\mathcal{R})$ and the orientational counterpart $g_6(\mathcal{R})$ on L. The spatial decay of these correlation functions is found to depend on L within the examined range. Nonetheless, the core physical picture remains unchanged. Specifically, the successive solid-to-hexatic and hexatic-to-liquid transitions reported previously persist and adhere to the KTHNY framework. Furthermore, the hexatic phase is identified at Pe $= 2.3$, which is slightly below the previously demarcated regime Pe $\in [2.35, 2.4]$ when $L = 100$, reinforcing the solidity of our findings.

Because the fundamental findings of our study have remained consistent, we have not enlarged the domain further for a more definitive insight. In fact, the rapidly growing computational cost renders the process prohibitively resource-intensive.

Tab. S1 Comparison of dipole-induced chain formation as observed in Ref. [48] and this study.

	Ref. [48]	This study
Source of dipole	Magnetic or electric	Hydrodynamic
Structure of chains	Head-to-tail connection (Fig. 5 of Ref. [48])	Side-by-side connection (Fig. S11C-D)
Features of dipolar interactions	Pairwise; short-ranged	Many-body; long-ranged

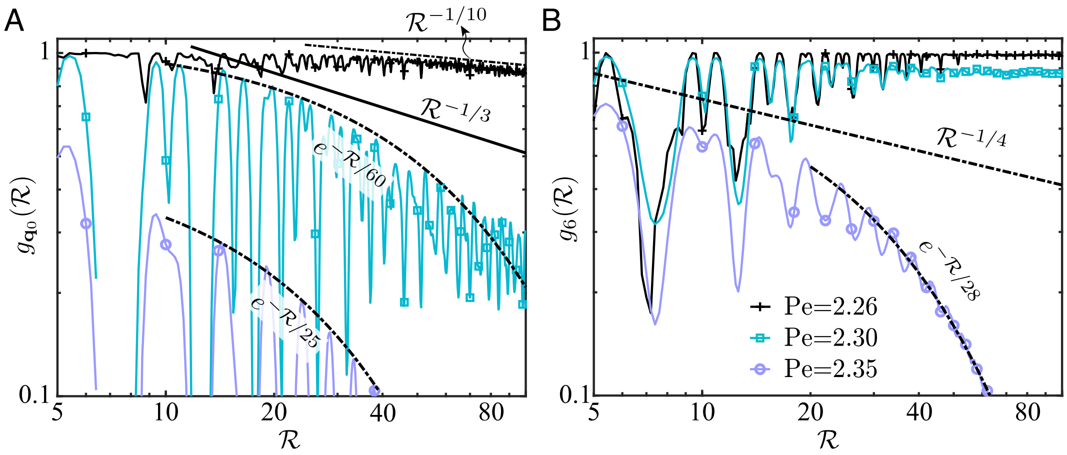

Fig. S13 : 2D melting of phoretic disks when $L = 200$ as compared to previously used $L = 100$ in Fig. 3 of the main article, with the area fraction $\phi = 0.12$ unchanged. A, translational order correlation function $g_{\mathbf{q}_0}(\mathcal{R})$ at $\mathrm{Pe} = [2.26, 2.3, 2.35]$. B, similar to A, but for the orientational order correlation function $g_6(\mathcal{R})$.

F. Transition to and active turbulence

When Pe = 5 and $\phi = 0.5$, a self-organized oscillatory active flow of disks develops, as demonstrated in the main article; its Fig. 4**a** captures an instant when the disks concentrate in the corners of the domain. Within the oscillation, the concentrated disk region periodically relocates between the corners and the center of the domain. For completeness, we present in Fig. S14 the respective configuration of disk accumulation in the center.

Fig. S15 showcases the Lagrangian characterization of vortical structures formed by disks, which corroborates the Eulerian counterpart illustrated in Fig. 5**b** of the main article.

1. *Effect of finite inertia on active turbulence*

As indicated in Fig. S4B, the effect of inertia Re = 0.5 on the propulsion of a single phoretic disk is weak. However, this indication for single swimmers does not conclusively demonstrate that this inertia level has negligible influence on the collective swimming dynamics.

To gain a more definitive insight, we have expanded our simulations to incorporate both smaller and larger Re values relative to our baseline setting of Re = 0.5. Specifically, we examine new cases with Re = 0.1, Re = 1, and Re = 2. To focus on the Re-dependence, a domain size of $L = 200$ is adopted for all the four cases. We conduct the comparative analysis, showing the instantaneous continuum flow field and the probability density function (PDF) of vortex sizes in Fig. S16, the PDF of the disks' velocity components and the longitudinal velocity differences in Fig. S17, as well as the kinetic energy spectrum in Fig. S18. As shown by Fig. S16 and Fig. S17, within the studied range of $\mathrm{Re} \in [0.1, 2]$, we do not observe qualitative differences in the continuum flow or the statistics of disks' velocities. Additionally, Fig. S18 indicates that the overall trend of the energy spectrum $E(\hat{q})$ remains consistent across varying Re. However, it is important to note that we do not assert the exact scaling exponents of $E(\hat{q})$ to be strictly independent of Re. Such a dependency or lack thereof is, in any case, of limited consequence.

2. *Resemblance with active nematics and polar active fluids*

The recent review [50] categorizes active turbulence into two types, based on whether the contributing entities exhibit polar or nematic behaviors. Interestingly, we find that the active turbulence of IPAs, which we study, might introduce a third category. This new category demonstrates distinct characteristics from both the former categories—it shares the unique oscillatory transitional scenario with polar active fluids [50, 51] and quali-

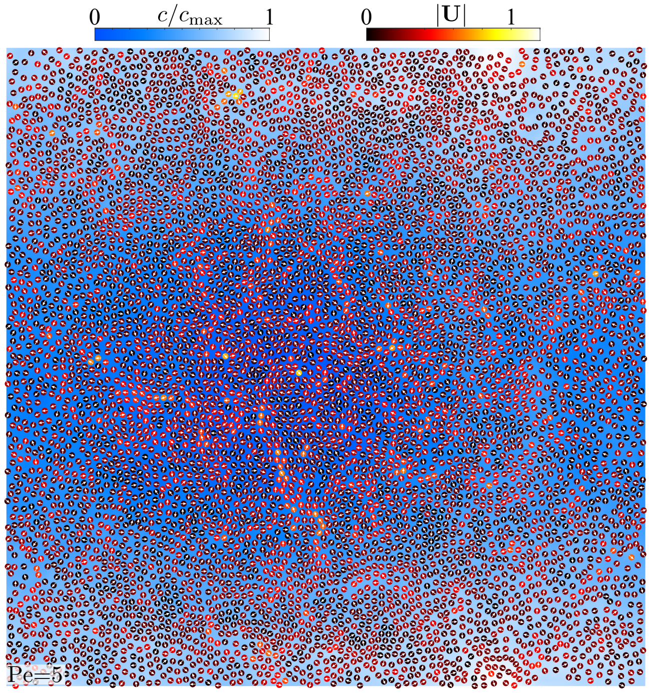

Fig. S14 : A snapshot of an oscillatory flow at Pe = 5 shows that disks concentrate in the center of the domain, contrasting with the corner concentration depicted in Fig. 4**a** of the main article. Here, $\phi = 0.5$.

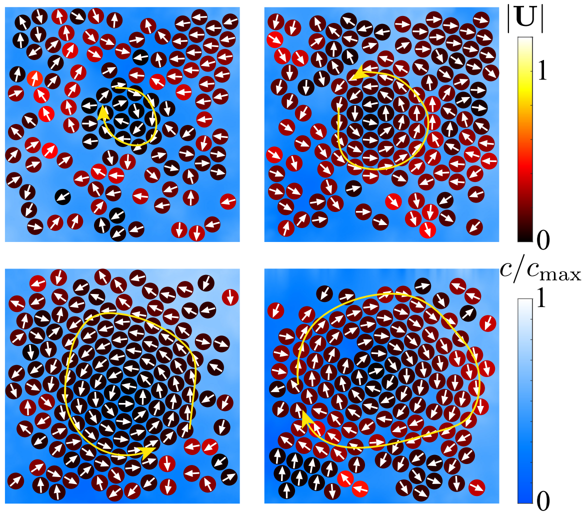

Fig. S15 : Lagrangian description of vortical structures formed by disks at Pe = 20, where $\phi = 0.5$ and $L = 200$. The edges of these vortexes are outlined by yellow curves.

tatively exhibits the scaling behavior of the energy spectrum typical of active nematics [52, 53]. A simple, though perhaps superficial, explanation for these observations could be attributed to the dual-state nature of an IPA. In its stable state, an IPA is stationary and thus apolar, similar to a nematic entity. However, when unstable, it behaves like a polar swimmer, akin to a Janus colloid or bacterium.

G. Clustering of phoretic disks

We note that clustering of active colloids has been reported before, *e.g.*, in Ref. [54]. Using Brownian dynamics simulations, the authors studied dilute suspensions of Janus-type phoretic colloids driven by their self-generated chemical fields, undergoing translational and rotational motion. Importantly, the study reproduces the experimentally observed dynamic clustering of Janus colloids, revealing significant clustering when the two types of motion lead to competing attractive and repulsive interactions, respectively. Here, we highlight several key differences between our work and Ref. [54] that may influence dynamic clustering:

- Polarity of swimmer: Ref. [54] considers Janus-like phoretic swimmers with inherent polarity, whereas we study isotropic phoretic swimmers that spontaneously develop a swimming orientation via instability. In our case, the polarity of a swimmer can change abruptly due to the influence of surrounding swimmers.

- Mechanism of attractive and repulsive interactions: In Ref. [54], the swimmer's response to the concentration gradient ∇c—via translational/rotational velocity—modulates the chemotaxis of swimmers. Translational motion is prescribed to induce attractive interactions, while rotational motion generates either attractive or repulsive interactions. In contrast, our study does not prescribe the kinematics or chemotactic response of our swimmers. Instead, it forms part of the solution, reflecting a Pe-dependent balance between chemical and hydrodynamic interactions. Notably, while our swimmers are chemo-repulsive, their dipolar flow produces hydrodynamic attraction as a signature of pushers. At low Pe values, the chemical repulsion leads to the formation of a Wigner crystal. When Pe sufficiently increases, the hydrodynamic attraction overcomes the chemical repulsion, causing the swimmers to dynamically chain together. Finally, at high Pe and area fractions, dynamic clustering emerges.

- Dry or wet active matter: Ref. [54] considers point swimmers without considering hydrodynamics. Instead, we account for full hydrodynamic and chemical interactions between finite-sized swimmers.

H. Reconciling two experimental observations on camphor surfers

In the main article, we present a comprehensive simulation study that harmonizes the findings of two sep-

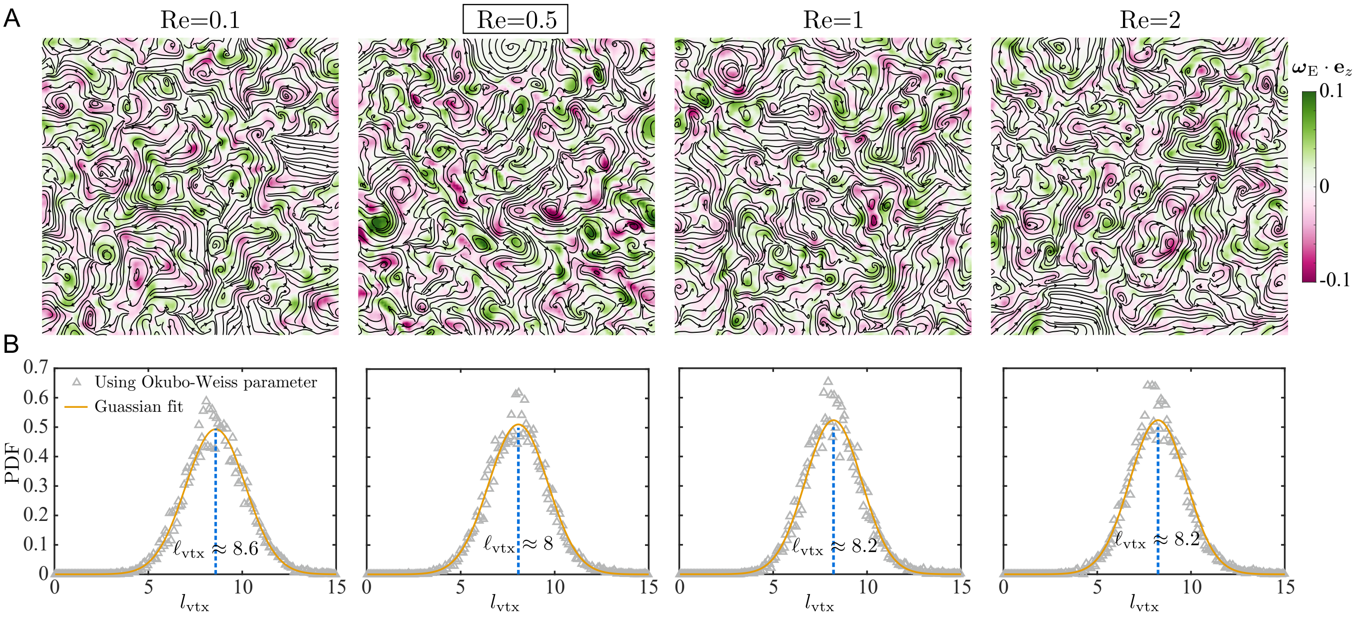

Fig. S16 : Influence of inertia, Re, on the continuum flow field by considering the disk suspension as a continuum active fluid. In addition to our baseline case Re = 0.5 demonstrated in the main article, new simulations for Re = 0.1, 1, and 2 have been conducted. Here, Pe = 20, $\phi = 0.5$, and $L = 200$. A, instantaneous streamlines and vorticity component $\boldsymbol{\omega}_{\mathrm{E}} \cdot \mathbf{e}_z$. B, size distribution of vortices identified using the Okubo-Weiss parameter.

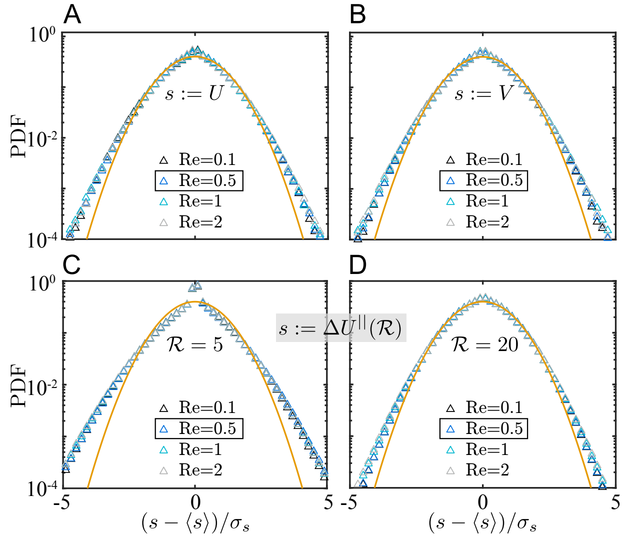

Fig. S17 : Similar to Fig. S16, while focusing on the statistics of disks' velocities. A and B, PDF of the disk velocity components U and V, respectively. C, PDF of the longitudinal component $\Delta U^{\|}$ of the velocity difference between two disks separated by a distance $\mathcal{R} = 5$. D, same as C, but for $\mathcal{R} = 20$. Here, $\langle s \rangle$ and σ_s represent the average and standard deviation of a random variable s, respectively. The curve corresponds to the unit-variance Gaussian function $1/\sqrt{2\pi}\exp(-s^2/2)$.

arate experiments on camphor surfers, which independently observed states of crystallization [27] and active turbulence [55]. The discrepancy between these observed phenomena, we suggest, is largely due to the different levels of phoretic activity, Pe, presented in the two experiments. We will detail this explanation further.

Both experiments employed camphor surfers of a disk shape, albeit with different dimensions. The disk diameter is 1 mm in Ref. [27], whereas it is 5 mm in Ref. [55]. Moreover, the height of disk is quite similar in both studies, specifically 500 and 600 μm respectively. Furthermore, both experiments followed the same protocol [56, 57] to fabricate the disks, which should ensure their comparable levels of chemical activity. Besides the disk itself, another factor that can influence its behavior is the depth of the subsurface fluid, which was set at 5 mm in Ref. [27] and at 10 mm in Ref. [55]. However, in both scenarios, these depths are considerably greater than three times the radius of the disk. As per the study [58], when the depth of the fluid exceeds this threshold, its influence on the disk's behavior becomes negligible. Collectively recalling the linear dependence of Pe on the diameter, we thus deduce that the Pe of the latter experiment is approximately five times that of the former one. This trend indeed qualitatively aligns with our predictions (Fig. 2 of the main article), wherein lower activity leads to crystallization and higher activity induces active turbulence.

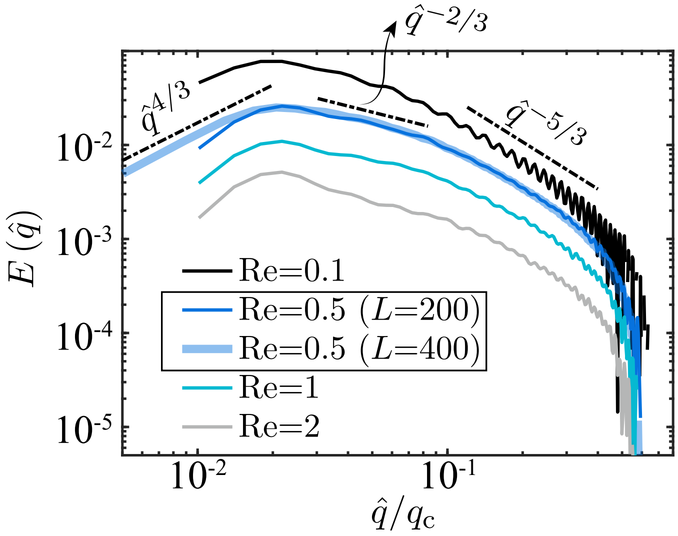

Fig. S18 : The effect of inertia Re on the energy spectrum $E(\hat{q})$ versus the modified wavenumber $\hat{q}$ (see Eq. (18)), which is vertically shifted for a better comparison. Here, $\phi = 0.5$ and $\mathrm{Pe} = 20$.

I. Isotropic phoretic agents (IPAs) in an unbounded domain

While we have focused on a periodic domain in this study, it is also worth noting potential scenarios involving multiple IPAs in an unbounded domain.

Though a single unbounded IPA achieves propulsion only via instability, this is not the case for multiple IPAs. As long as $\mathrm{Pe} > 0$, a pair of identical IPAs will consistently maintain a finite translational velocity and have zero rotational velocity. This occurs due to the disruption of their spherical or circular symmetry resulting from their mutual interaction, as highlighted in Ref. [59].

When there are more than two IPAs ($n > 2$), they will engage in translational motion, however, the disappearance of their rotational motion is not a certainty but rather contingent on their initial configuration. Despite the challenge in deciphering their behavior in a general scenario when $n > 2$, we can make a qualitative prediction for three particles ($n = 3$): their rotational motion ceases when their centers align in a straight line or are equidistant (essentially forming a regular triangle), whereas under other conditions, rotation may typically ensue.

In the unbounded case as discussed above, the particles will progressively move away from each other over time due to the inherent chemical repulsion. Ultimately, each particle will reach a degree of separation substantial enough to essentially restore an isolated state. At this juncture, the particle motion will once again depend on Pe.

Unlike the unbounded case characterized by a zero area or volume fraction, the periodic domain features a finite fraction—a configuration we have adopted in our work. In such a setting, our simulations show that multiple disks initiate movement irrespective of Pe. However, given a sufficiently low Pe, indicative of suppressed instability, these disks will eventually self-organize into a stationary crystalline state.

SUPPLEMENTARY REFERENCES

[1] A. Okubo, "Horizontal dispersion of floatable particles in the vicinity of velocity singularities such as convergences," *Deep-Sea Res. Oceanogr. Abstr.*, vol. 17, no. 3, pp. 445–454, 1970.

[2] J. Weiss, "The dynamics of enstrophy transfer in two-dimensional hydrodynamics," *Physica D*, vol. 48, no. 2-3, pp. 273–294, 1991.

[3] M. Theers, E. Westphal, K. Qi, R. G. Winkler, and G. Gompper, "Clustering of microswimmers: interplay of shape and hydrodynamics," *Soft Matter*, vol. 14, no. 42, pp. 8590–8603, 2018.

[4] E. Bonabeau, L. Dagorn, and P. Fréon, "Scaling in animal group-size distributions," *Proc. Natl. Acad. Sci. USA*, vol. 96, no. 8, pp. 4472–4477, 1999.

[5] D. Nishiguchi and M. Sano, "Mesoscopic turbulence and local order in Janus particles self-propelling under an ac electric field," *Phys. Rev. E*, vol. 92, no. 5, p. 052309, 2015.

[6] K. Qi, E. Westphal, G. Gompper, and R. G. Winkler, "Emergence of active turbulence in microswimmer suspensions due to active hydrodynamic stress and volume exclusion," *Commun. Phys.*, vol. 5, no. 1, p. 49, 2022.

[7] A. W. Zantop and H. Stark, "Emergent collective dynamics of pusher and puller squirmer rods: swarming, clustering, and turbulence," *Soft Matter*, vol. 18, no. 33, pp. 6179–6191, 2022.

[8] S. Chen and G. D. Doolen, "Lattice Boltzmann method for fluid flows," *Annu. Rev. Fluid Mech.*, vol. 30, no. 1, pp. 329–364, 1998.

[9] C. S. Peskin, "Flow patterns around heart valves: a numerical method," *J. Comput. Phys.*, vol. 10, no. 2, pp. 252–271, 1972.

[10] R. Mittal and G. Iaccarino, "Immersed boundary methods," *Annu. Rev. Fluid Mech.*, vol. 37, pp. 239–261, 2005.

[11] A. Scagliarini and I. Pagonabarraga, "Unravelling the role of phoretic and hydrodynamic interactions in active colloidal suspensions," *Soft Matter*, vol. 16, no. 38, pp. 8893–8903, 2020.

[12] G. Gompper, T. Ihle, D. M. Kroll, and R. G. Winkler, "Multi-particle collision dynamics: A particle-based mesoscale simulation approach to the hydrodynamics of complex fluids," *Advanced Computer Simulation Approaches for Soft Matter Sciences III*, pp. 1–87, 2009.

[13] M.-J. Huang, J. Schofield, and R. Kapral, "Chemotactic and hydrodynamic effects on collective dynamics of self-diffusiophoretic Janus motors," *New J. Phys.*, vol. 19, no. 12, p. 125003, 2017.

[14] E. Lauga and T. R. Powers, "The hydrodynamics of swimming microorganisms," *Rep. Prog. Phys.*, vol. 72, no. 9, p. 096601, 2009.

[15] M. Jiang and Z. Liu, "A boundary thickening-based direct forcing immersed boundary method for fully resolved simulation of particle-laden flows," *J. Comput. Phys.*, vol. 390, pp. 203–231, 2019.

[16] M. Jiang, J. Li, and Z. Liu, "A simple and efficient parallel immersed boundary-lattice Boltzmann method for fully resolved simulations of incompressible settling suspensions," *Comput. Fluids*, vol. 237, p. 105322, 2022.

[17] W. W. Ren, C. Shu, J. Wu, and W. M. Yang, "Boundary condition-enforced immersed boundary method for thermal flow problems with Dirichlet temperature condition and its applications," *Comput. Fluids*, vol. 57, pp. 40–51, 2012.

[18] S. K. Kang and Y. A. Hassan, "A comparative study of direct-forcing immersed boundary-lattice Boltzmann methods for stationary complex boundaries," *Int. J. Numer. Methods Fluids*, vol. 66, no. 9, pp. 1132–1158, 2011.

[19] J. R. Blake, "A spherical envelope approach to ciliary propulsion," *J. Fluid Mech.*, vol. 46, no. 1, pp. 199–208, 1971.

[20] M. J. Lighthill, "On the squirming motion of nearly spherical deformable bodies through liquids at very small Reynolds numbers," *Commun. Pure Appl. Anal.*, vol. 5, no. 2, pp. 109–118, 1952.

[21] G. Zhu and L. Zhu, "Self-propulsion of an elliptical phoretic disk emitting solute uniformly," *J. Fluid Mech.*, vol. 974, p. A57, 2023.

[22] W.-F. Hu, T.-S. Lin, S. Rafai, and C. Misbah, "Chaotic swimming of phoretic particles," *Phys. Rev. Lett.*, vol. 123, no. 23, p. 238004, 2019.

[23] D. Nie and J. Lin, "Simulation of sedimentation of two spheres with different densities in a square tube," *J. Fluid Mech.*, vol. 896, 2020.

[24] J. Bialké, T. Speck, and H. Löwen, "Crystallization in a dense suspension of self-propelled particles," *Phys. Rev. Lett.*, vol. 108, no. 16, p. 168301, 2012.

[25] J. Palacci, S. Sacanna, A. P. Steinberg, D. J. Pine, and P. M. Chaikin, "Living crystals of light-activated colloidal surfers," *Science*, vol. 339, no. 6122, pp. 936–940, 2013.

[26] A. P. Petroff, X.-L. Wu, and A. Libchaber, "Fast-moving bacteria self-organize into active two-dimensional crystals of rotating cells," *Phys. Rev. Lett.*, vol. 114, no. 15, p. 158102, 2015.

[27] S. Soh, K. J. M. Bishop, and B. A. Grzybowski, "Dynamic self-assembly in ensembles of camphor boats," *J. Phys. Chem. B*, vol. 112, no. 35, pp. 10848–10853, 2008.

[28] G. Briand and O. Dauchot, "Crystallization of self-propelled hard discs," *Phys. Rev. Lett.*, vol. 117, no. 9, p. 098004, 2016.

[29] R. Singh and R. Adhikari, "Universal hydrodynamic mechanisms for crystallization in active colloidal suspensions," *Phys. Rev. Lett.*, vol. 117, no. 22, p. 228002, 2016.

[30] J. U. Klamser, S. C. Kapfer, and W. Krauth, "Thermodynamic phases in two-dimensional active matter," *Nat. Commun.*, vol. 9, no. 1, pp. 1–8, 2018.

[31] B. Kichatov, A. Korshunov, V. Sudakov, V. Gubernov, I. Yakovenko, and A. Kiverin, "Crystallization of active emulsion," *Langmuir*, vol. 37, no. 18, pp. 5691–5698, 2021.

[32] C. Kittel and P. McEuen, *Introduction to Solid State Physics*. John Wiley & Sons, 2018.

[33] A. R. West, *Solid State Chemistry and Its Applications*. John Wiley & Sons, 2022.

[34] E. Wigner, "On the interaction of electrons in metals," *Phys. Rev.*, vol. 46, no. 11, p. 1002, 1934.

[35] E. Wigner, "Effects of the electron interaction on the energy levels of electrons in metals," *Trans. Faraday Soc.*, vol. 34, pp. 678–685, 1938.

[36] H. Li, S. Li, E. C. Regan, D. Wang, W. Zhao, S. Kahn, K. Yumigeta, M. Blei, T. Taniguchi, K. Watanabe, S. Tongay, A. Zettl, M. F. Crommie, and F. Wang, "Imaging two-dimensional generalized Wigner crystals," *Nature*, vol. 597, no. 7878, pp. 650–654, 2021.

[37] P. Illien, R. Golestanian, and A. Sen, "'Fuelled' motion: phoretic motility and collective behaviour of active col-

loids," *Chem. Soc. Rev.*, vol. 46, no. 18, pp. 5508–5518, 2017.

[38] R. Golestanian, "Phoretic active matter," *Active Matter and Nonequilibrium Statistical Physics, Lecture Notes of the Les Houches Summer School*, vol. 112, pp. 230–293, 2019.

[39] B. Liebchen and A. K. Mukhopadhyay, "Interactions in active colloids," *J. Phys.: Condens. Matter*, vol. 34, no. 8, p. 083002, 2021.

[40] K. Lippera, M. Benzaquen, and S. Michelin, "Alignment and scattering of colliding active droplets," *Soft Matter*, vol. 17, no. 2, pp. 365–375, 2021.

[41] B. V. Hokmabad, J. Agudo-Canalejo, S. Saha, R. Golestanian, and C. C. Maass, "Chemotactic self-caging in active emulsions," *Proc. Natl. Acad. Sci. USA*, vol. 119, no. 24, p. e2122269119, 2022.

[42] B. V. Hokmabad, A. Nishide, P. Ramesh, C. Krüger, and C. C. Maass, "Spontaneously rotating clusters of active droplets," *Soft Matter*, vol. 18, no. 14, pp. 2731–2741, 2022.

[43] K. Lippera, M. Morozov, M. Benzaquen, and S. Michelin, "Collisions and rebounds of chemically active droplets," *J. Fluid Mech.*, vol. 886, 2020.

[44] S. Michelin, E. Lauga, and D. Bartolo, "Spontaneous autophoretic motion of isotropic particles," *Phys. Fluids*, vol. 25, no. 6, p. 061701, 2013.

[45] A. Zöttl and H. Stark, "Emergent behavior in active colloids," *J. Phys. Condens. Matter*, vol. 28, no. 25, p. 253001, 2016.

[46] S. Thutupalli, R. Seemann, and S. Herminghaus, "Swarming behavior of simple model squirmers," *New J. Phys.*, vol. 13, no. 7, p. 073021, 2011.

[47] S. Thutupalli, D. Geyer, R. Singh, R. Adhikari, and H. A. Stone, "Flow-induced phase separation of active particles is controlled by boundary conditions," *Proc. Natl. Acad. Sci. USA*, vol. 115, no. 21, pp. 5403–5408, 2018.

[48] H. Schmidle, C. K. Hall, O. D. Velev, and S. H. L. Klapp, "Phase diagram of two-dimensional systems of dipole-like colloids," *Soft Matter*, vol. 8, no. 5, pp. 1521–1531, 2012.

[49] S. H. L. Klapp, "Collective dynamics of dipolar and multipolar colloids: From passive to active systems," *Curr. Opin. Colloid Interface Sci.*, vol. 21, pp. 76–85, 2016.

[50] R. Alert, J. Casademunt, and J.-F. Joanny, "Active turbulence," *Annu. Rev. Condens. Matter Phys.*, vol. 13, no. 10.1146, 2022.

[51] L. Giomi and M. C. Marchetti, "Polar patterns in active fluids," *Soft Matter*, vol. 8, no. 1, pp. 129–139, 2012.

[52] R. Alert, J.-F. Joanny, and J. Casademunt, "Universal scaling of active nematic turbulence," *Nat. Phys.*, vol. 16, no. 6, pp. 682–688, 2020.

[53] B. Martínez-Prat, R. Alert, F. Meng, J. Ignés-Mullol, J.-F. Joanny, J. Casademunt, R. Golestanian, and F. Sagués, "Scaling regimes of active turbulence with external dissipation," *Phys. Rev. X*, vol. 11, no. 3, p. 031065, 2021.

[54] O. Pohl and H. Stark, "Dynamic clustering and chemotactic collapse of self-phoretic active particles," *Phys. Rev. Lett.*, vol. 112, no. 23, p. 238303, 2014.

[55] M. Bourgoin, R. Kervil, C. Cottin-Bizonne, F. Raynal, R. Volk, and C. Ybert, "Kolmogorovian active turbulence of a sparse assembly of interacting Marangoni surfers," *Phys. Rev. X*, vol. 10, no. 2, p. 021065, 2020.

[56] C. J. Campbell, E. Baker, M. Fialkowski, and B. A. Grzybowski, "Arrays of microlenses of complex shapes prepared by reaction-diffusion in thin films of ionically doped gels," *Appl. Phys. Lett.*, vol. 85, no. 11, pp. 1871–1873, 2004.

[57] S. K. Smoukov, K. J. M. Bishop, R. Klajn, C. J. Campbell, and B. A. Grzybowski, "Cutting into solids with micropatterned gels," *Adv. Mater.*, vol. 17, no. 11, pp. 1361–1365, 2005.

[58] D. Boniface, C. Cottin-Bizonne, R. Kervil, C. Ybert, and F. Detcheverry, "Self-propulsion of symmetric chemically active particles: Point-source model and experiments on camphor disks," *Phys. Rev. E*, vol. 99, no. 6, p. 062605, 2019.

[59] B. Nasouri and R. Golestanian, "Exact phoretic interaction of two chemically active particles," *Phys. Rev. Lett.*, vol. 124, no. 16, p. 168003, 2020.
