# Supplementary material for: Shaping active matter from crystalline solids to active turbulence
Source: Nat Commun. 2024 Apr 3;15:2874. doi: 10.1038/s41467-024-46520-4 (PMC11258367; doi:10.1038/s41467-024-46520-4)
Supplement: Supplementary file 3 — Description of Additional Supplementary Files [file 41467_2024_46520_MOESM3_ESM.pdf]

# Description of Additional Supplementary Files for ‘Shaping active matter from crystalline solids to active turbulence’

Qianhong Yang ,[1,*] Maoqiang Jiang ,[2,1,*] Francesco Picano ,[3] and Lailai Zhu [1,†]

[1] *Department of Mechanical Engineering, National University of Singapore, 117575, Singapore*
[2] *School of Naval Architecture, Ocean and Energy Power Engineering, Wuhan University of Technology, Wuhan, Hubei, 430063, PR China*
[3] *Department of Industrial Engineering and CISAS “G. Colombo”, University of Padova, Padova, 35122, Italy*

## SUPPLEMENTARY VIDEOS 1-11

- Filename: Supplementary Video 1
  Description: Self-organization into a crystalline solid phase at a Péclet number $\mathrm{Pe} = 2$ and area fraction $\phi = 0.12$.
- Filename: Supplementary Video 2
  Description: Emergence of a gas-like phase with disks forming dynamic chains when $\mathrm{Pe} = 3$ and $\phi = 0.12$.
- Filename: Supplementary Video 3
  Description: Formation of a liquid phase when $\mathrm{Pe} = 2.5$ and $\phi = 0.12$.
- Filename: Supplementary Video 4
  Description: Disassociation of a bound dislocation pair into two free dislocations when a hexatic phase is identified at $\mathrm{Pe} = 2.4$ and $\phi = 0.12$. A portion of the domain is shown here.
- Filename: Supplementary Video 5
  Description: Certain dislocations unbind into isolated disclinations and they coexist in this liquid phase at $\mathrm{Pe} = 2.5$ and $\phi = 0.12$. A portion of the domain is shown here.
- Filename: Supplementary Video 6
  Description: An oscillatory instability occurs in the form of large-scale wave motion at $\mathrm{Pe} = 5$, where $\phi = 0.5$ and the domain size $L = 200$.
- Filename: Supplementary Video 7
  Description: A transitional scenario characterized by emerging disk clusters that breakdown the wave pattern. Here, $\mathrm{Pe} = 10$, $\phi = 0.5$, and $L = 200$.
- Filename: Supplementary Video 8
  Description: Active turbulence featuring both vortical structures and clusters formed by disks. Here, $\mathrm{Pe} = 20$, $\phi = 0.5$, and $L = 200$.
- Filename: Supplementary Video 9
  Description: Crossing and reflecting trajectories of two interacting phoretic disks of $\mathrm{Pe} = 2.5$ as discussed in the Supplementary Information (SI).
- Filename: Supplementary Video 10
  Description: Unlike Supplementary Video 9, two disks of $\mathrm{Pe} = 2.5$ can also form a stable bound pair swimming in parallel as discussed in the SI.
- Filename: Supplementary Video 11
  Description: A three-disk chain executes circular or straight trajectories intermittently as discussed in the SI, where $\mathrm{Pe} = 20$.

* These two authors contributed equally
† lailai_zhu@nus.edu.sg
